# Comparative Genomic and Phenotypic Characterization of Pathogenic and Non-Pathogenic Strains of *Xanthomonas arboricola* Reveals Insights into the Infection Process of Bacterial Spot Disease of Stone Fruits

**DOI:** 10.1371/journal.pone.0161977

**Published:** 2016-08-29

**Authors:** Jerson Garita-Cambronero, Ana Palacio-Bielsa, María M. López, Jaime Cubero

**Affiliations:** 1 Instituto Nacional de Investigación y Tecnología Agraria y Alimentaria (INIA), Madrid, Spain; 2 Centro de Investigación y Tecnología Agroalimentaria de Aragón, Instituto Agroalimentario de Aragón-IA2 - (CITA-Universidad de Zaragoza), Zaragoza, Spain; 3 Instituto Valenciano de Investigaciones Agrarias (IVIA), Moncada, Valencia, Spain; Oklahoma State University, UNITED STATES

## Abstract

*Xanthomonas arboricola* pv. *pruni* is the causal agent of bacterial spot disease of stone fruits, a quarantinable pathogen in several areas worldwide, including the European Union. In order to develop efficient control methods for this disease, it is necessary to improve the understanding of the key determinants associated with host restriction, colonization and the development of pathogenesis. After an initial characterization, by multilocus sequence analysis, of 15 strains of *X*. *arboricola* isolated from *Prunus*, one strain did not group into the pathovar *pruni* or into other pathovars of this species and therefore it was identified and defined as a *X*. *arboricola* pv. *pruni* look-a-like. This non-pathogenic strain and two typical strains of *X*. *arboricola* pv. *pruni* were selected for a whole genome and phenotype comparative analysis in features associated with the pathogenesis process in *Xanthomonas*. Comparative analysis among these bacterial strains isolated from *Prunus* spp. and the inclusion of 15 publicly available genome sequences from other pathogenic and non-pathogenic strains of *X*. *arboricola* revealed variations in the phenotype associated with variations in the profiles of TonB-dependent transporters, sensors of the two-component regulatory system, methyl accepting chemotaxis proteins, components of the flagella and the type IV pilus, as well as in the repertoire of cell-wall degrading enzymes and the components of the type III secretion system and related effectors. These variations provide a global overview of those mechanisms that could be associated with the development of bacterial spot disease. Additionally, it pointed out some features that might influence the host specificity and the variable virulence observed in *X*. *arboricola*.

## Introduction

*Xanthomonas arboricola* [1] is a species of Gram negative, rod-shaped bacteria exclusively associated with plants. Most of the strains of this species cause diseases on several herbaceous and woody plants of agricultural interest. Beside these, some other strains have been identified as non-pathogenic, saprophytic or opportunistic pathogens. Based on the host specialization of the pathogenic strains, nine pathovars have been recently proposed [2].

*X*. *arboricola* pv. *pruni*, causal agent of bacterial spot disease on at least 13 species of the genus *Prunus*, is considered one of the most economically important pathovars within *X*. *arboricola*. This pathogen, which is classified as a quarantinable organism in the European Union, causes damages on leaves, fruits, twigs, branches and trunks of the trees [3]. Damages in 25–75% of the peach fruits in orchards in the USA have been reported [4].

Previous molecular typing analysis of a selection of strains isolated from *Prunus* has demonstrated the low diversity on *X*. *arboricola* pv. *pruni*, which forms a monophyletic group comprised of a unique clonal complex regardless of the host, the continent or the year of isolation, corresponding to a pandemic lineage that stays as a monomorphic group during evolution [2,5].

Next generation sequencing approaches have provided valuable information related to the genomics of the genus *Xanthomonas*, allowing understanding of the role of several pathogenic factors as well as the key determinants of bacterial adaptation and host restriction [6]. Since 2012, genome sequencing projects have started for *X*. *arboricola*, generating at least one complete genome and 15 draft genome sequences, comprising meaningful information for understanding pathogenesis in this species and for the improvement of diagnostic tools addressed to disease prevention. Comparative genomic studies in *X*. *arboricola* are attracting increased interest in this species and have started to bring important results. For instance, in a study on *X*. *arboricola* pv. *juglandis*, causal agent of bacterial blight on walnut (*Juglans* spp.), a group of non-pathogenic strains isolated from *J*. *regia* were identified and classified as phylogenetically distant from the pathogenic strains. Complete genome analysis of these atypical strains revealed differences from pathogenic strains in features related to the initial steps of bacterial infection, such as those connected to structural components of the flagellar system, non-fimbrial adhesins and chemosensors. Furthermore, the profile of type III effectors was correlated with the capacity to produce disease on walnut [7,8].

Previous studies of plant-pathogen interaction in other models such as *X*. *citri* [9,10], *X*. *campestris* [11–13] and *X*. *oryzae* [14,15], have demonstrated that the study of phenotypic and genotypic features associated with bacterial sensing and attachment, chemotaxis, motility, xanthan production, biofilm organization, the metabolism of carbon sources, as well as secretion of virulence factors, were basic for improving the knowledge of the bacterial ability to penetrate the plant tissue and to cause disease in a specific host range of plants.

The general genome features of two *X*. *arboricola* pv. *pruni* pathogenic strains, isolated from almond (*Prunus amygdalus*, syn. *P*. *dulcis*) and Japanese plum (*Prunus salicina*), as well as one of a non-pathogenic strain isolated from Santa Lucía SL-64 rootstock (*Prunus mahaleb*) were described in a previous work [16,17]. Herein, our goal was to associate the genomic content of these xanthomonads with the phenotypic features of *X*. *arboricola* pv. *pruni* that causes bacterial spot of stone fruits. Differences in key aspects associated with bacterial sensing, motility, attachment and secretion of cell-wall degrading enzymes as well as type III effectors were determined in pathogenic and non-pathogenic strains of *X*. *arboricola*.

## Results

### Molecular characterization using multilocus sequence analysis (MLSA), genome based phylogeny and genome comparison

As an initial approach to characterize a group of strains phenotypically similar to *Xanthomonas* isolated from *Prunus* in Spain, one *Xanthomonas* strain isolated from asymptomatic leaves of *P*. *mahaleb* (strain CITA 44), and 14 *Xanthomonas* strains isolated from *Prunus* spp. with symptoms of bacterial spot disease, were typed at four loci (*dnaK*, *fyuA*, *gyrB* and *rpoD*) in order to determine their taxonomic position [18] (Table 1).

**Table 1:** *Xanthomonas arboricola* strains used in this study.

Strain^a^	Pathovar	Host	Year of isolation	Origin Country (province)
*X*. *arboricola*				
CITA 44		*Prunus mahaleb* (rootstock Santa Lucía SL-64)	2009	Spain (Aragón)
ICMP 1488^¥^	*celebensis*	*Musa acuminate*	1960	New Zealand
CFBP 1846*	*corylina*	*Corylus avellana*	1975	France
ICMP 5726^¥^	*corylina*	*Corylus maxima*	1939	United States
ICMP 35^¥^	*juglandis*	*Juglans regia*	1956	New Zealand
IVIA 2113*	*juglandis*	*Juglans regia*	2010	Spain (Badajoz)
CFBP 3123^PT^*	*populi*	*Populus* x *euramericana* cv Robusta	1979	Netherlands
10.0343*	*pruni*	*Prunus laurocerasus* cv. Caucasica	2010	Netherlands
10.0400*	*pruni*	*Prunus laurocerasus* cv. Grüner Teppich	2010	Netherlands
10.0439*	*pruni*	*Prunus laurocerasus* cv. Zabeliana	2010	Netherlands
CFBP 3894^PT^*	*pruni*	*Prunus salicina*	1953	New Zealand
CFBP 5530*	*pruni*	*Prunus persica*	1989	Italy
CFBP 5724*	*pruni*	*Prunus amygdalus*	Unknown	United States
CITA 4	*pruni*	*Prunus persica* cv. Catherine	2004	Spain (Valencia)
CITA 9	*pruni*	*Prunus persica* cv. Merrill O'Henry	2008	Spain (Aragón)
CITA 11	*pruni*	*Prunus persica* cv. Richard Lady	2008	Spain (Aragón)
CITA 33	*pruni*	*Prunus amygdalus* cv. Guara	2009	Spain (Aragón)
CITA 46	*pruni*	*Prunus persica* cv. Summer Lady	2009	Spain (Navarra)
CITA 70^**^	*pruni*	*P*. *persica* x *P*. *amygdalus* (rootstock Garnem (GxN15)	2010	Italy
IVIA 2626.1	*pruni*	*Prunus salicina* cv. Fortuna	2002	Spain (Badajoz)
IVIA 2647-1-2	*pruni*	*Prunus salicina* cv. Larry Ann	2002	Spain (Badajoz)
IVIA 2647-3-1	*pruni*	*Prunus salicina* cv. Friar	2002	Spain (Badajoz)
IVIA 2826–1	*pruni*	*Prunus salicina* cv. Anne Gold	2003	Spain (Valencia)
IVIA 2832–10	*pruni*	*Prunus salicina* cv. Angeleno	2003	Spain (Valencia)
IVIA 3161–2	*pruni*	*Prunus amygdalus* cv. Rumbeta	2006	Spain (Alicante)
IVIA 3162	*pruni*	*Prunus amygdalus* cv. Rumbeta	2006	Spain (Alicante)
IVIA 3487–1	*pruni*	*Prunus salicina*	2008	Spain (Aragón)

^a^ CITA, Centro de Investigación y Tecnología Agroalimentaria de Aragón, Zaragoza, Spain; ICMP, International Collection of Microorganisms from Plants, Auckland, New Zealand; CFBP, Collection Française de Bactéries Phytopathogénes, Angers, France; IVIA, Instituto Valenciano de Investigaciones Agrarias, Valencia, Spain.

^PT^ Pathotype strains.

* Strains characterized previously as *Xanthomonas arboricola* and used in this study as control during the molecular characterization.

_**_ Interception in a Spanish nursey (Aragón) of plants produced in an Italian nursery.

^¥^ Sequences from these type strains were obtained from a previous work [18], and used as reference for the MLSA in this paper.

**Fig 1:**
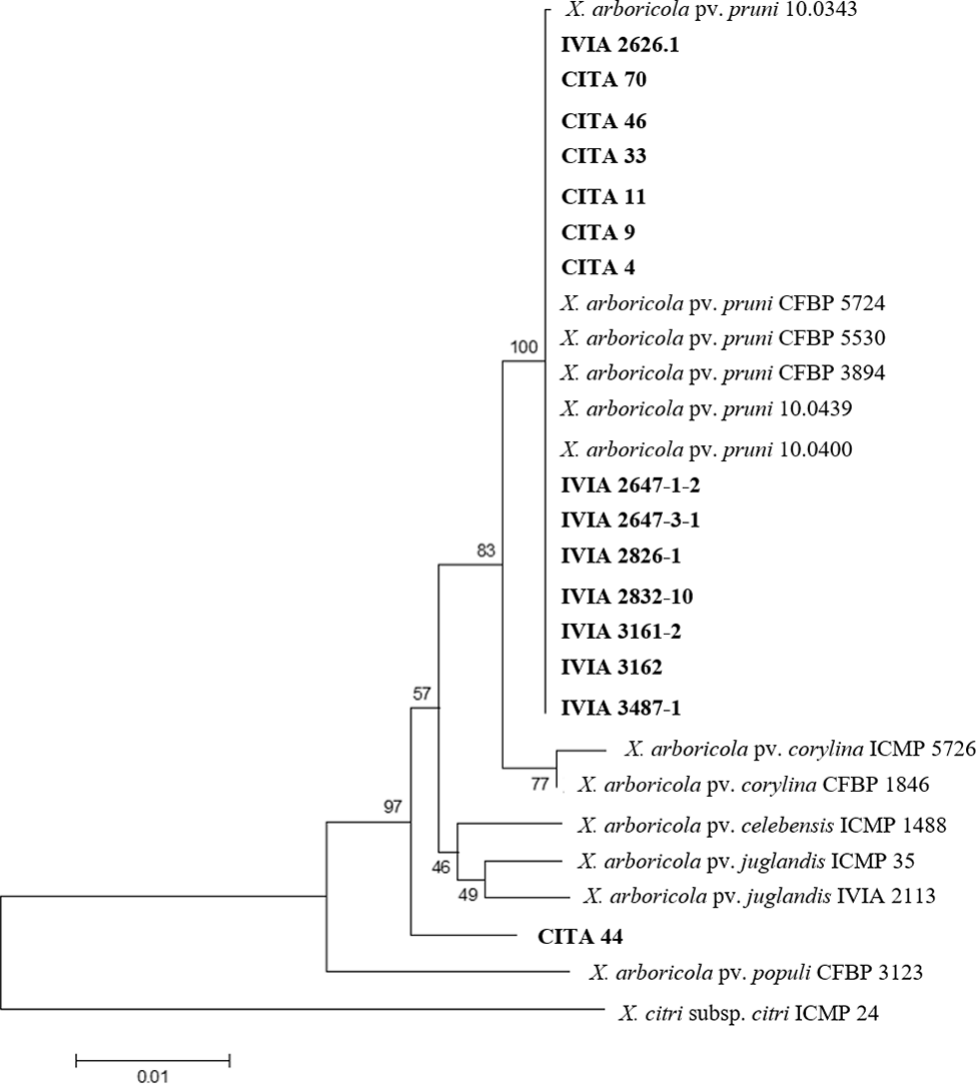
Maximum likelihood tree of concatenated nucleotide sequences for partial sequences of the genes *dnaK*, *fyuA*, *gyrB* and *rpoD* of selected strains of *X*. *arboricola* isolated from *Prunus*. Target strains are in bold. For comparative purposes other *X*. *arboricola* strains are included. *X*. *citri* subsp. *citri* strain ICMP 24 was considered as outgroup. Bootstrap values (1,000 replicates) are indicated below and above the branches.

Analysis of the concatenated sequence (2,858 nucleotide positions) revealed a percent of similarity from 96.86 to 100% within the group encompassed by six *X*. *arboricola* reference strains and the 15 strains isolated from *Prunus* spp. Despite this, strain CITA 44, according to the maximum likelihood analysis, could not be consistently associated with any of the reference strains (Fig 1). The remaining 14 strains isolated from symptomatic hosts were clustered into a group with all the reference strains of *X*. *arboricola* pv. *pruni*. Comparative analysis between these two intra-specific groups revealed a total of 43 nucleotide variations in the concatenated sequence of CITA 44 (11 variable nucleotide sites for *dnaK*, nine for *fyuA*, 16 for *gyrB* and seven for *rpoD* genes), or a sequence similarity of 98.49% between this strain and all the other *X*. *arboricola* isolated from *Prunus*. These nucleotide changes could be associated with silent mutations, due to the fact that the translated amino acid sequence of each locus showed a 100% of sequence coverage and 99–100% of sequence identity to several strains of *X*. *arboricola* according to the Blastp results.

**Fig 2:**
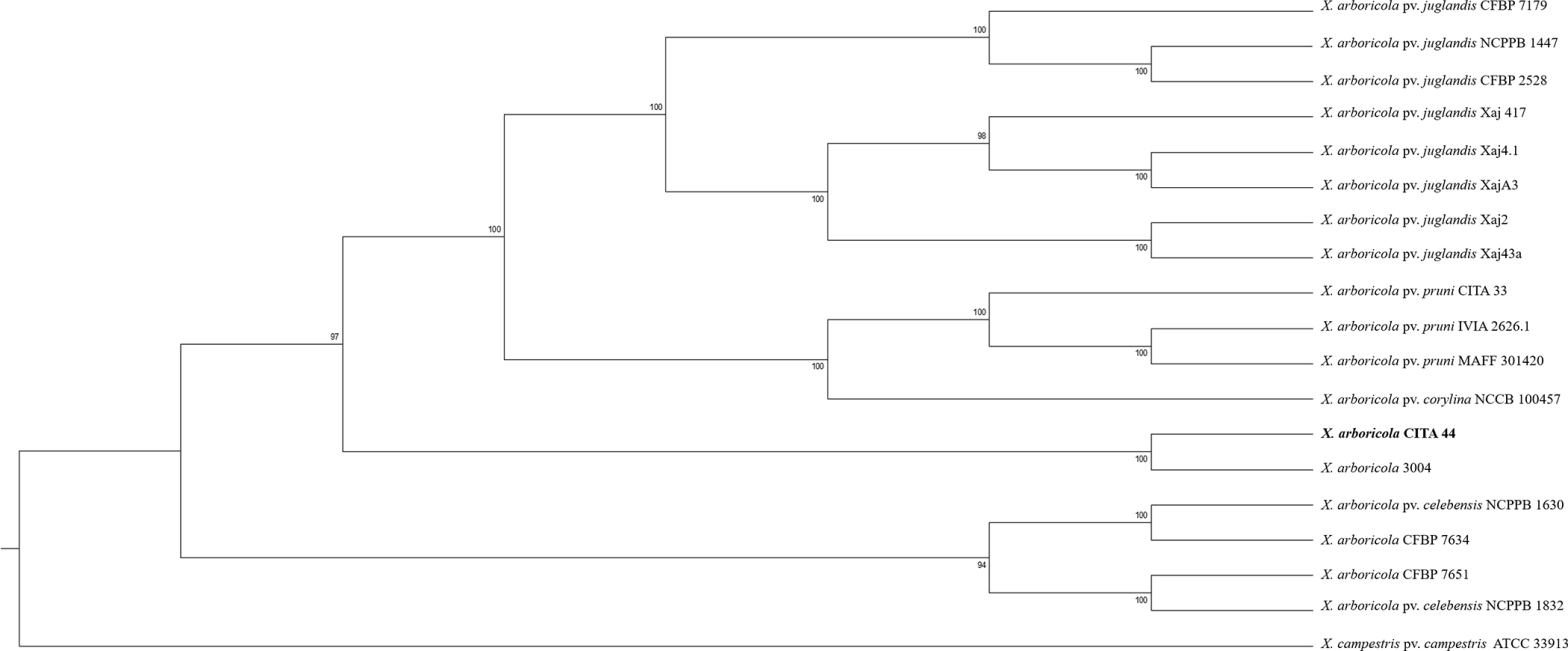
Phylogenetic analysis of 18 strains of *X*. *arboricola* based on the core genome sequence of the species (2,525 CDS). Sequences were aligned using MAFFT and Maximum likelihood analysis was carried out using RaxML. Bootstrap values (1,000 replicates) are presented above or below the branches. The strain of *X*. *campestris* pv. *campestris* ATCC 33913 was used as an outgroup.

Phylogenetic analysis of the 18 *X*. *arboricola* strains, based on the core genome sequence (S1 and S2 Tables), showed that the three strains that cause disease on stone fruits and almond (CITA 33, IVIA 2626.1 and MAFF 301420) clustered according to the pathovar classification, as observed previously using the multilocus sequence analysis (MLSA) approach. In the same manner, strain CITA 44 did not cluster with the other xanthomonads isolated from *Prunus* nor to any of the well-established pathovars described for *X*. *arboricola*. Instead, CITA 44 was included in a clade along with the strain 3004 of *X*. *arboricola*, recently described as the causal agent of disease on barley (*Hordeum vulgare*) [19] (Fig 2).

**Fig 3:**
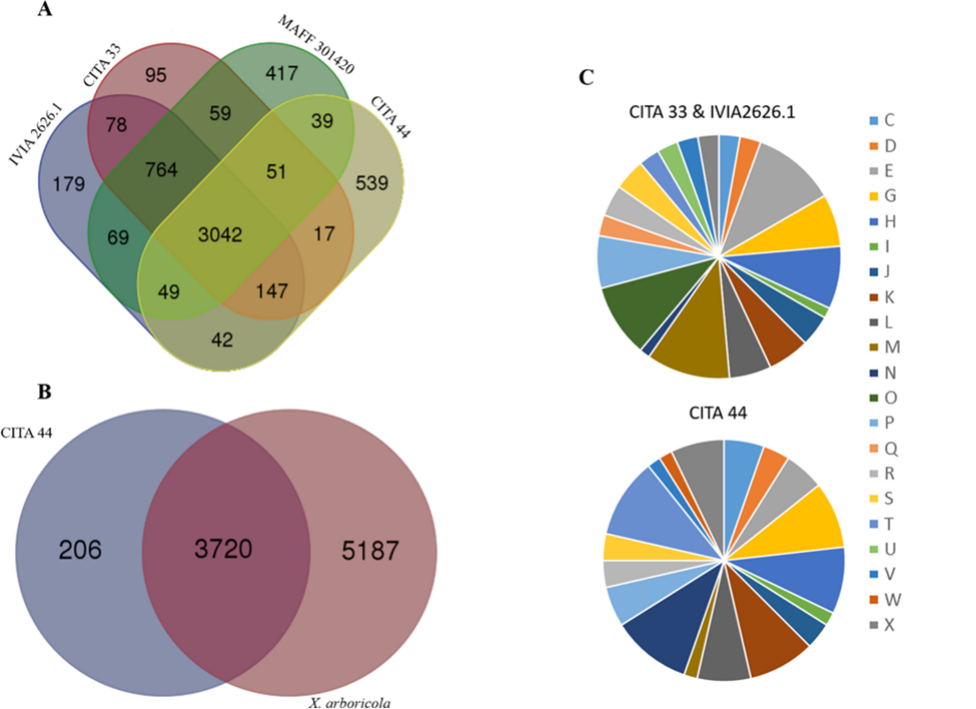
Venn diagram representing the CDS shared among *X*. *arboricola*. CDS shared by *Prunus*-pathogenic strains and CITA 44 (A), CDS shared by CITA 44 and *X*. *arboricola* (B). The distribution of the unique CDS found in in the draft genome sequences of CITA 44, CITA 33 and IVIA 2626.1 that have been classified into the COGs functional categories are also represented (C). Different letters represent the standardized code of the COGs functional categories.

After automatic annotation of 18 genome sequences publicly available of *X*. *arboricola* [7,16,17,19–23], 3,927 protein coding sequences (CDS) were predicted for CITA 44, whereas in the draft genome sequence of the other *X*. *arboricola* strains that did not represent a well-established pathovar (strains 3004, CFBP 7634 and CFBP 7651), a total of 5,221 different CDS were determined. For the strains of the pathovars *celebensis*, *juglandis* and *pruni*, 4,485; 5,700 and 5,048 CDS were predicted, respectively (S1 Table). The core genome sequence of the analyzed strains was comprised by at least 2,525 CDS (S2 Table). CITA 44 shared 3,387 CDS with the three strains of the pathovar *pruni* (Fig 3A, S1 Fig) and 3,720 CDS with all the strains of *X*. *arboricola* (Fig 3B). This strain shared the highest number of CDS with strain 3004 of *X*. *arboricola* (3,485 CDS), not classified in any pathovar, whereas the lowest number of CDS was shared with the strain MAFF 301420 of the pathovar *pruni* (3,181 CDS). Unique CDS for strains CITA 44, CITA 33 and IVIA 2626.1 were predicted. CITA 44 showed 206 exclusive CDS, 55 of which were classified into 18 clusters of orthologous groups (COG) functional categories, being predominantly those related to cell motility and carbohydrate transport and metabolism. Signal peptide cleavage sites and transmembrane helices were predicted only in 19 and 33 CDS, respectively. In the case of the *X*. *arboricola* pv. *pruni* strains, 149 unique CDS were found, 54 of them were classified into 20 COG functional categories, with a predominant presence of CDS related to cell wall/membrane biogenesis and amino acids transport and metabolism. Additionally, 17 CDS showed signal peptide cleavage sites and transmembrane helices were predicted in 33 CDS (Fig 3C, S3 Table).

### Carbon sources utilization and chemotaxis profile

**Fig 4:**
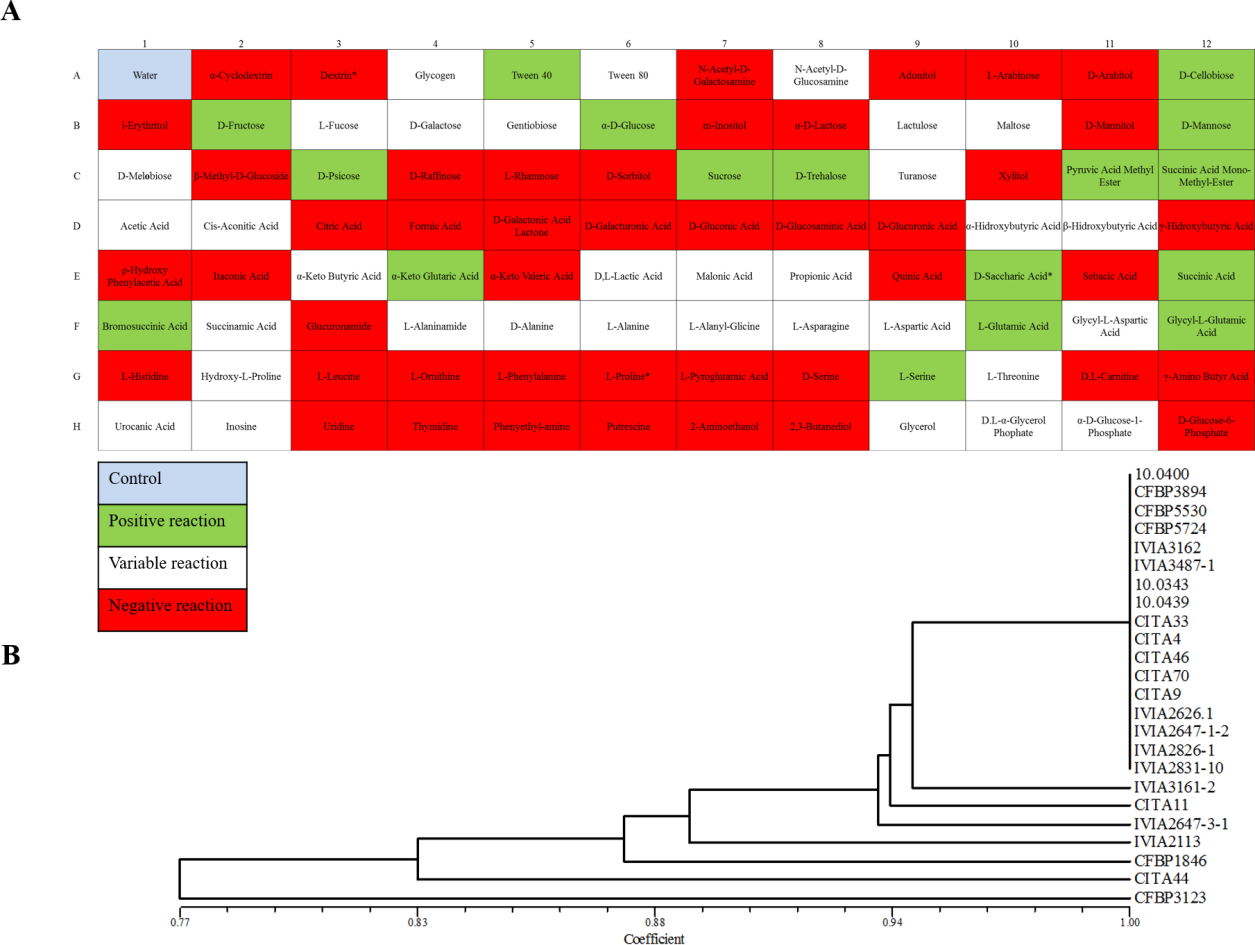
Carbon compounds utilization profile of *X*. *arboricola*. Biolog GN2 profile for CITA 44 (A), comparative cluster analysis including carbon compounds profile from *X*. *arboricola* isolates (B). Cluster analysis was constructed based on 63 informative substrates. Data were computed using the UPGMA model. Reliability of the tree was determined by the cophenetic correlation index (*r* > 0.98).

The profile of carbon sources metabolized by CITA 44 was determined using the BIOLOG GN2 microplate system and compared to that obtained from the strains of the pathovars *corylina*, *juglandis*, *populi* and *pruni* (Table 1). Seventeen carbon sources compounds over 95 were utilized by CITA 44, meanwhile 45 carbon sources were not utilized and 33 showed variable reactions and, consequently, were considered as not informative. On the other hand, the profile observed in 20 strains isolated from *Prunus* and identified as *X*. *arboricola* pv. *pruni*, as well as the patterns observed in the remaining strains of *X*. *arboricola*, were different compared to CITA 44, as represented in the dendrogram obtained from the similarity analysis (Fig 4). All the strains of the pathovar *pruni*, unlike CITA 44, were able to metabolize dextrin and proline and unable to metabolize D-saccharic acid.

In order to determine the chemotactic effect of 18 carbon compounds on CITA 44 and on two reference strains of *X*. *arboricola* pv. *pruni*, a microtiter plate chemotaxis assay was conducted [24]. Strain CITA 44 was attracted only by serine (200 mM), and repelled by citric acid (10 mM), galacturonic acid (10 mM), glucuronic acid (10 mM), arginine (10 mM), cysteine (10 mM) and galactose (10 mM). No chemotactic activity was recorded toward the remaining compounds assayed. Strains CFBP 5530 and CITA 33 showed a different chemotactic pattern compared to CITA 44. The first two strains were attracted to glycerol (2%), maltose (10 mM) and serine (10 mM and 200 mM), and repelled by citric acid (10 mM), galacturonic acid (10 mM), glucuronic acid (10 mM) and cysteine (10 mM). Detailed chemotactic patterns for each strain are shown in Table 2.

**Table 2:** Chemotactic profile of 18 carbon compounds of three representative strains of *Xanthomonas arboricola* isolated from *Prunus* spp.

Carbon compounds	CFBP 5530	CITA 33	CITA 44
Alanine 10 mM	+	0	0
Alanine 250 mM	+	0	0
Arginine 10 mM	0	-	-
Arginine 100 mM	+	0	0
Citric acid 10 mM	-	-	-
Cysteine 10 mM	-	-	-
Fructuose 10 mM	+	0	0
Galactose 10 mM	+	0	-
Galacturonic acid 10 mM	-	-	-
Glucose 10 mM	0	0	0
Glucuronic acid 10 mM	-	-	-
Glycerol 0.2%	0	0	0
Glycerol 2%	+	+	0
Leucine 10 mM	0	0	0
Leucine 150 mM	0	+	0
Maltose 10 mM	+	+	0
Mannitol 0.2%	0	0	0
Serine 10 mM	+	+	0
Serine 200 mM	+	+	+
Sodium citrate 10 mM	+	0	0
Succinic acid 10 mM	0	0	0
Sucrose 10 mM	0	+	0
Xylose 10 mM	0	0	0

+, Chemotactic activity

-, Chemorepellent activity

0, No chemotactic activity

In addition to the phenotypic variants observed in the features mentioned above, genome comparison also revealed variants in the profile of the environmental receptors studied within this species. *X*. *arboricola* strains harbored 14 of the 28 TonB-dependent transporters (TBDTs) analyzed [25] (S4 Table), and from these, only two homologs to the TBDTs encoded by the loci XAC3620 (outer membrane receptor FepA) and XCC3595 (ferric pseudobactin receptor), described in *X*. *citri* and *X*. *campestris*, respectively, were shared by all the *X*. *arboricola* strains. All strains isolated from *Prunus* spp., both pathogenic and non-pathogenic, harbored eight TBDTs. The presence of homologous sequences to the TonB-dependent receptor loci XCC1719 and XAC3077, and the absence of XCC0304 and XCC2867 in the strains of *X*. *arboricola* pv. *pruni*, differentiated them from the non-pathogenic strain CITA 44. The distribution of the TBDTs revealed that none of these variations was unique for those strains that inhabit the *Prunus* hosts. Nevertheless, the TBDT *cirA* (XAC3077) was found only in those strains that caused disease on stone fruits and Turkish hazel (*Corylus colurna*).

**Fig 5:**
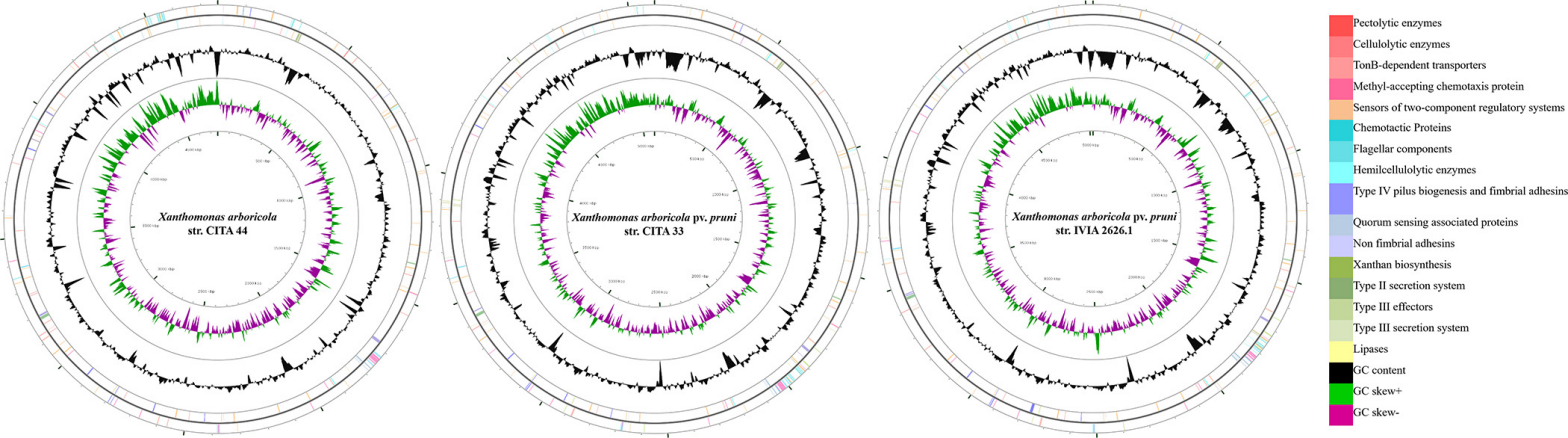
Graphical circular representation of the CDS associated with pathogenesis in *X*. *arboricola* isolated from *Prunus*. The contigs of the draft genome sequence of strains CITA 33, CITA 44 and IVIA 2626.1 were arranged by Mauve [26], using the genome sequence of *X*. *arboricola* pv. *juglandis* Xaj 417 as the reference. The circular map was constructed using CGview. From outside to center: CDS on forward strand, CDS on reverse strand, GC content and GC skew.

Regarding the distribution of sensors of two-component regulatory system (STCRS) [25], 58 orthologous CDS of 86 genes previously described in *Xanthomonas* were found in *X*. *arboricola*. All the analyzed strains shared 44 STCRS (S4 Table), and CITA 33, CITA 44 and IVIA 2626.1 have 53 STCRS in common. Homologous CDS to a diguanylate cyclase of *X*. *citri* (XAC2804) was only present in the pathogenic strains, meanwhile homologues of four STCRS (XAC1819, XAC2804, XAC0136 and XAC1345) were only found in CITA 44. Additionally, as compared to other *X*. *arboricola* strains, CITA 33 and IVIA 2626.1 presented two unique STCRS homologous to the loci XAC0136 and XAC1345 of *X*. *citri*, respectively (Fig 5).

Out of 26 methyl-accepting chemotaxis proteins (MCPs) previously described [25], 22 were found in the genome sequences of *X*. *arboricola* (S4 Table). The number of MCPs varied from 16 in strain CFBP 7634 to 22 in strains 3004, CFBP 7651 and NCPPB 1630; all the *X*. *arboricola* strains shared a MCPs pattern composed by 13 genes (S4 Table). *Prunus*-associated strains shared 20 MCPs, pathogenic strains differed from CITA 44 in the existence of a CDS orthologous to the MCP XCV1933 and the absence of the MCP XCV1938, both described in *X*. *campestris* pv. *vesicatoria*. No MCPs could be detected as specific for a particular host plant, however, orthologous CDS for three MCPs described in *X*. *citri* and *X*. *campestris* pv. *vesicatoria* (XAC3768, XCV1952 and XCV1938) were absent only in the strain that causes disease on Turkish hazel (S4 Table).

*X*. *arboricola* genome sequences harbored 15 to 17 genes homologous to the chemotactic related *che* genes [25] (S4 Table). Pathogenic strains of *Prunus* had 17 homologous CDS for these genes, and CITA 44 had 16 CDS. CITA 44 did not show homologous CDS to the locus XAC2447 (*cheW*) described in *X*. *citri*, which was present in all the remaining genome sequences of *X*. *arboricola* (Fig 5).

### Flagella, fimbrial and non-fimbrial adhesins associated with motility and attachment in *X*. *arboricola*

**Fig 6:**
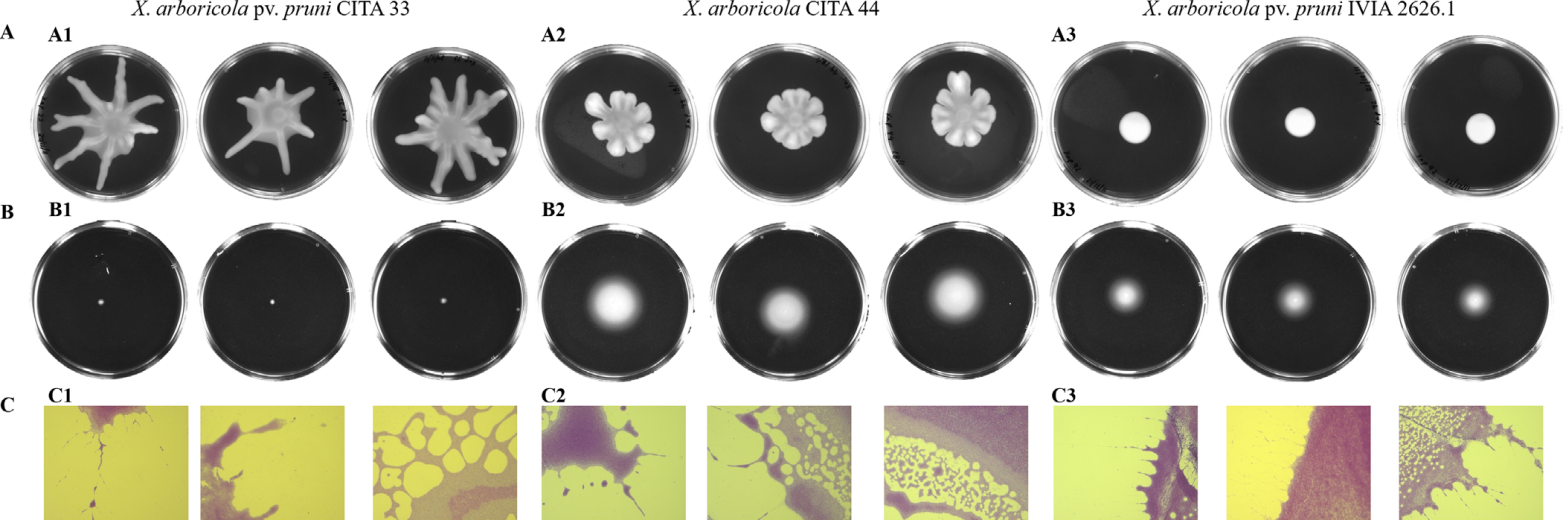
Flagella and type IV pilus mediated motility on semisolid and solid surfaces in some representative strains of *Xanthomonas arboricola*. Swarming dendritic (A1 and A2) and circular (A3) phenotypes were determined on PYM 0.5% agar plates after 24 hpi. Variable swimming phenotypes (B1, B2 and B3) observed after 72 hpi on MMA 0.3% agar plates. Twitching type motility (C1, C2 and C3) was observed after 72 hpi on polyestyrene plate surface after inoculation through PYM 1.5% agar (40X). Each motility assay was conducted in three independent experiments with three replicates.

Swarming motility was evaluated in all the 21 *X*. *arboricola* strains isolated from *Prunus* (Table 3). Two kinds of swarmer colony phenotype were observed. Dendritic pattern, encompassed by several tendrils that extended away from a central colony, was observed in CITA 44 as well as in other 13 strains of the pathovar *pruni* after 12–15 hours post inoculation (hpi). Despite this, CITA 44 showed some unique characteristic such as the presence of shorter and wider tendrils in the swarming colony (Fig 6A). The second colony morphology was characterized by a circular shape shown by seven strains of *X*. *arboricola* pv. *pruni* (Fig 6A). Light and electron microscopy observations discarded the existence of hyper-flagellated bacterial cells in the swarming colony for all strains, including CITA 44 (S2 Fig).

**Table 3:** Motility profiles of 21 strains of *Xanthomonas arboricola* on semisolid surfaces.

Strain	Pathovar	Swarming^α^	Surfactant activity^β^	Swimming^γ^
100.343	*pruni*	17.15±6.22*	3.06±0.42	0.58±0.10
100.400	*pruni*	4.91±2.82*	3.64±0.74	0.55±0.05
100.439	*pruni*	30.25±14.84*	1.63±0.42	0.55±0.05
CFBP 3894	*pruni*	4.30±1,14°	1.59±0.48	0.31±0.03
CFBP 5530	*pruni*	3.32±0,66°	2.87±0.32	0.65±0.13
CFBP 5724	*pruni*	4.78±1.70°	2.18±0.33	1.25±0.05
CITA 4	*pruni*	13.47±7.04*	6.62±0.93	0.50±0.05
CITA 9	*pruni*	17.78±7.66*	1.94±0.56	0.65±0.05
CITA 11	*pruni*	4.24±1.54*	4.22±1.22	0.55±0.05
CITA 33	*pruni*	18.72±9.62*	2.80±0.66	0.60±0.05
CITA 44	-	13.42±6.02*	3.69±0.54	2.42±0.10
CITA 46	*pruni*	22.16±11.91*	8.54±0.16	0.50±0.05
CITA 70	*pruni*	15.30±4.33*	5.44±0.79	0.52±0.08
IVIA 2626.1	*pruni*	2.75±0.83°	4.44±0.76	0.30±0.05
IVIA 2647-1-2	*pruni*	2.32±0.42°	3.94±0.78	0.55±0.05
IVIA 2647-3-1	*pruni*	23.55±14.67*	3.91±0.64	0.40±0.05
IVIA 2826–1	*pruni*	17.98±8.23*	3.55±0.45	0.45±0.05
IVIA 2832–10	*pruni*	21.45±7.41*	2.94±0.52	0.55±0.05
IVIA 3161–2	*pruni*	3.15±1.04°	2.97±0.73	0.45±0.05
IVIA 3162	*pruni*	2.71±0.55°	0.52±0.10	0.78±0.08
IVIA 3487–1	*pruni*	5.89±2.39*	11.98±0.89	0.50±0.00

^α^ Mean ± SD of the area (cm^2^) covered by the swarmer colony on PYM 0.5% agar plates after 24 hpi

* Dendritic swarming phenotypes shown by the analyzed strains.

° Circular swarming phenotypes shown by the analyzed strains.

^β^ Mean ± SD of the ratio between the radius of the bright halo and the area of the colony observed 24 hpi after atomizing mineral oil over the bacterial colony.

^γ^ Mean ± SD of the radius (cm) of the swimming colony after 72 hpi on MMA 0.3% agar plates.

Surfactant activity was evaluated for the 21 strains mentioned above according to the atomized oil assay [27]. A bright halo, associated with a change in the surface tension, was observed around the colonies of all the strains tested. Surfactant production, estimated as the ratio between the radius of the bright halo and the area of the colony, was variable among strains (Table 3). CITA 44 showed a mean ratio of 3.69 ± 0.54 (mean ± standard deviation), meanwhile strains of the pathovar *pruni* showed a mean ratio of 3.94 ± 2.62. Strains IVIA 3162 and IVIA 3847–1 showed mean ratios of 0.52 ± 0.10 and 11.98 ± 0.89, being the lowest and the highest activity identified, respectively (Table 3).

The swimming ability of these strains was also assayed on 0.3% agar MMA plates. After inoculation, all the strains showed a circular and turbid growth around the inoculating point (Fig 6B). This activity, recorded as the radius of the bacterial colony, was variable among the strains (Table 3). CITA 44 presented the highest activity with an average of the radius of the halo of 2.42 ± 0.10, meanwhile the mean for pathogenic strains was 0.65 ± 0.46, with a maximum value of 1.25 ± 0.05 for CFBP 5724 and a minimum value of 0.30 ± 0.05 for IVIA 2626.1.

Twitching motility, which is carried out by type IV pilus [28], was observed in all the strains on the plastic surface of the culture plate, after crystal violet staining (Fig 6C). Microscopic observation at the edge of the colony revealed a twitching zone composed by bacterial rafts moving away from the colony, creating a concentric pattern preceded by a lattice like network and microcolonies.

Genome analysis performed to characterize the presence of the major structural components of the two motility and adherence structures described above [25,29], revealed that 33 (*X*. *arboricola* 3004 and *X*. *arboricola* pv. *corylina*) to 35 (*X*. *arboricola* pv. *pruni* and *X*. *arboricola* CFBP 7634 and CFBP 7651) flagellar components were found in *X*. *arboricola*. The genomes in these species shared 29 CDS (S4 Table). The strains isolated from *Prunus* presented 34 (CITA 44) and 35 (CITA 33 and IVIA 2626.1) structural components; CITA 44 did not show homologous sequence to *fliD* (XAC1974), which is present in all the analyzed strains (Fig 5, S4 Table). Despite the similar pattern of flagellar components observed among the *X*. *arboricola* strains, variants in the amino acid sequence of the flagellin protein were observed between pathogenic strains (CFBP 7179, CITA 33, IVIA 2626.1, MAFF 301420 and NCCB 100457), which showed the identical protein WP_039814449.1, and the non-pathogenic or low-pathogenic strains (3004, CFBP 7634, CFBP 7651, NCPPB 1630 and CITA 44), which showed the identical protein WP_024939608.1. Both proteins showed 354 identical amino acids of a total of 399 (pairwise identity of 88.7%). A remarkable difference, associated with the potential to infect in other xanthomonads, is the substitution of aspartic acid by valine in the amino acid position 43 of the N-terminal region of the flagellin gene in the pathovars *corylina*, *juglandis* and *pruni* (S3 Fig).

Regarding the structural and regulatory components of the type IV pilus, 22 out of 31 CDS homologous to the components described in *X*. *citri* subsp. *citri* strain 306 [30] were found in the nine genome sequences of *X*. *arboricola*; 16 of these CDS were shared by all the strains (S4 Table). The other *Prunus-*associated strains presented the same components, including CITA 44. The remaining strains of *X*. *arboricola* showed a similar pattern, with the exception of NCPPB 1630, CFBP 7634 and CFBP 7651, which did not have homologous sequence to the locus XAC0259 of *X*. *citri*. No sequence associated with *pilF* was observed in strain CFBP 7634 and none homologues of *pilQ* was found in strain 3004 (S4 Table).

Regarding the presence of the genes associated with type IV pilus, that are present in most of the *Xanthomonas* species, the cluster *pilB*, *C*, *D*, *R*, *S* was found in all the strains, as well as the cluster that encodes the minor pilins, *pilE*, *V*, *W*, *X*, *Y1* and *fimT*, that showed a sequence identity lower than 80.0%. Presence of *pilE*, *pilV*, *pilY1* and *fimT* was variable among the strains, meanwhile *pilW* and *pilX* were found in all the genome sequences. Prepilin *pilA* was found in all the strains of *X*. *arboricola*, despite its identity to the locus XAC3505 of *X*. *citri* was lower than 80.0% (S4 Table).

In addition to the fimbrial adhesins described above, the repertoire of non-fimbrial adhesins [25] of *X*. *arboricola* comprised five genes (S4 Table). All the strains shared homologous CDS to *panB* (XAC1816), *yapH* (XAC2151) and *xadA* (XCV3670) of *X*. *citri* and *X*. *campestris* pv. *vesicatoria*. *Prunus*-pathogenic strains harbored an adhesin homologous to the locus XAC3672 of *X*. *citri* which was absent in CITA44 (Fig 5; S4 Table). Finally, the hemagglutinin encoded by the locus XAC444 of *X*. *citri* was found only in those strains that were able to colonize hosts of the genus *Corylus*, *Junglans* and *Prunus*.

### Pathogenicity tests and genomic components associated with late stages of infection in *Prunus*-associated strains

**Fig 7:**
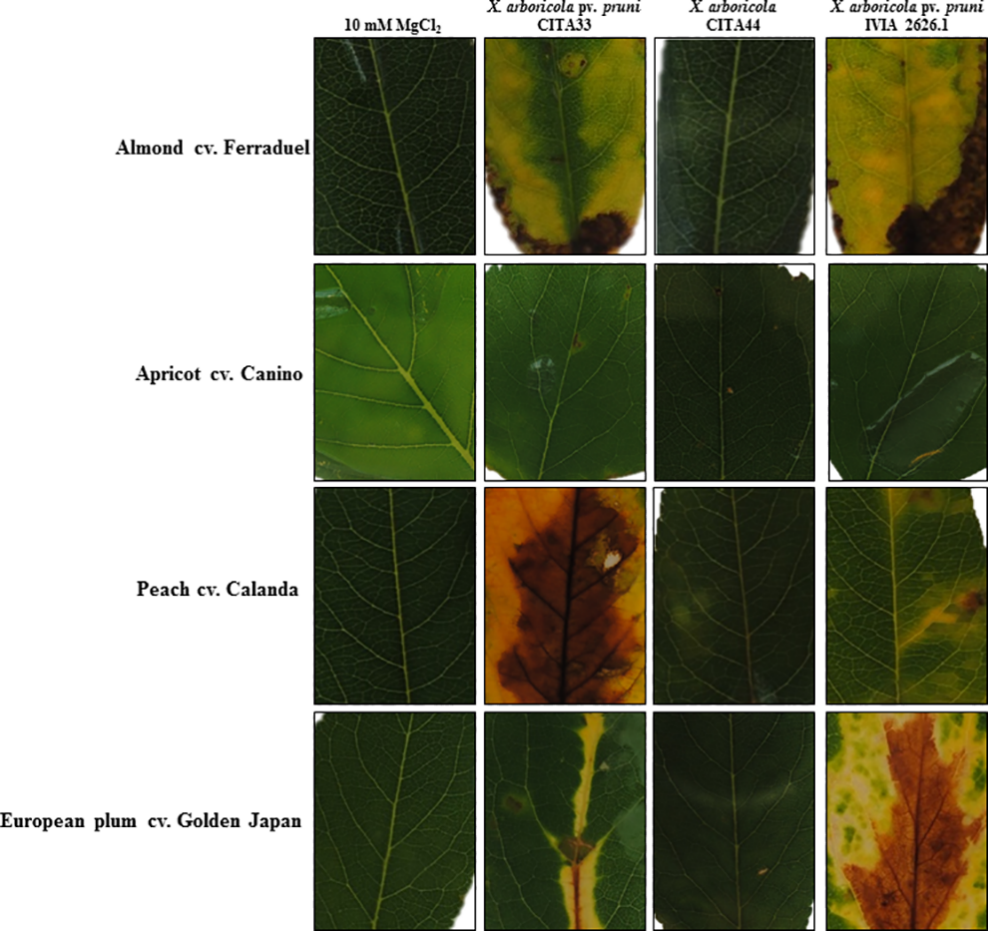
Schematic representation of bacterial spot symptoms observed on almond, apricot, peach and European plum 28 dpi. Detached leaves were inoculated with a sterile cotton swap damped with 1x10^8^ CFU/mL of bacteria or with 10 mM MgCl_2_ as negative control and maintained on 0.5% water agar plates.

Detached leaf assay was carried out by inoculating 21 *X*. *arboricola* strains, isolated from *Prunus* spp., on almond (cv. Ferraduel), apricot (cv. Canino), peach (cv. Calanda) and European plum (cv. Golden Japan). CITA 44 did not cause bacterial spot disease symptoms on almond, apricot, peach or plum 28 days post inoculation (dpi). Significant differences (*p* < 0.05) in the virulence among strains of the pathovar *pruni* were shown (Table 4). These strains were able to induce disease symptoms on almond, peach and European plum but none of them caused clear disease symptoms on apricot. Representatives of this assay are shown in Fig 7.

**Table 4:** Differences in the virulence of 24 *Xanthomonas arboricola* strains isolated from *Prunus* on detached leaves of almond, apricot, peach and European plum.

Strain	Pathovar	Almond^α^	Apricot	Peach	European plum
10.0343	*pruni*	69.21 ± 12.97_b_^DEF^	0_a_^A^	79.27 ± 14.13_b_^DE^	65.00 ± 20.94_b_^C^
10.0439	*pruni*	55.04± 13.21_b_^BCDEF^	0_a_^A^	69.09 ± 35.54_b_^CDE^	65.23 ± 13.10_b_^C^
10.0400	*pruni*	60.59 ± 7.17_b_^CDEF^	0_a_^A^	62.82 ± 26.55_b_^CDE^	57.35 ± 26.97_b_^BC^
CFBP 3894	*pruni*	54.11 ± 13.57_c_^BCDEF^	0_a_^A^	32.94 ± 29.19_bc_^BC^	24.12 ± 33.64_ab_^ABC^
CFBP 5530	*pruni*	24.74 ± 20.37_b_^ABC^	0_a_^A^	76.70 ± 14.45_c_^CDE^	57.23 ± 22.960_c_^BC^
CFBP 5724	*pruni*	61.65 ± 15.72_b_^CDEF^	0_a_^A^	60.23 ± 16.91_b_^CDE^	46.38 ± 29.31_b_^BC^
CITA 4	*pruni*	36.36 ± 27.88_b_^BCDE^	0_a_^A^	52.80 ± 34.04_b_^BCDE^	40.92 ± 31.23_b_^BC^
CITA 9	*pruni*	63.09 ± 13.47_b_^DEF^	0_a_^A^	50.28 ± 32.30_b_^BCDE^	42.36 ± 19.17_b_^BC^
CITA 11	*pruni*	60.52 ± 18.77_b_^CDEF^	0_a_^A^	48.18 ± 29.78_b_^BCDE^	44.95 ± 20.58_b_^BC^
CITA 33	*pruni*	56.30 ± 15.37_b_^BCDEF^	0_a_^A^	72.96 ± 22.19_b_^CDE^	56.87 ± 25.41_b_^BC^
CITA 44	-	0_a_^A^	0_a_^A^	0_a_^A^	0_a_^A^
CITA 46	*pruni*	65.93 ± 11.94_c_^DEF^	0_a_^A^	77.90 ± 20.09_c_^DE^	39.55 ± 22.71_b_^BC^
CITA 70	*pruni*	61.62 ± 14.56_b_^CDEF^	0_a_^A^	42.99 ± 28.09_b_^BCDE^	50.83 ± 20.71_b_^BC^
IVIA 2647-1-2	*pruni*	51.74 ± 15.80_b_^BCDEF^	0_a_^A^	73.05 ± 19.87_b_^CDE^	55.21 ± 24.10_b_^BC^
IVIA 2647-3-1	*pruni*	74.41 ± 22.75_c_^EF^	0.03 ± 0.08_a_^A^	69.38 ± 20.59_c_^CDE^	47.17 ± 18.42_b_^BC^
IVIA 2826–1	*pruni*	57.87 ± 16.05_c_^BCDEF^	0.60 ± 1.67_a_^A^	60.52 ± 26.40_c_^CDE^	33.11 ± 18.42_b_^ABC^
IVIA 2832–10	*pruni*	65.07 ± 16.01_b_^DEF^	0_a_^A^	81.49 ± 13.81_c_^E^	53.32 ± 14.05_b_^BC^
IVIA 3161–2	*pruni*	71.72 ± 16.47_b_^DEF^	0_a_^A^	75.29 ± 18.70_b_^CDE^	40.92 ± 33.80_b_^BC^
IVIA 3162	*pruni*	54.62 ± 14.67_bc_^BCDEF^	0_a_^A^	70.60 ± 28.90_c_^CDE^	41.97 ± 28.26_b_^BC^
IVIA 3847–1	*pruni*	52.98 ± 33.83_b_^BCDEF^	0_a_^A^	69.61 ± 12.97_b_^CDE^	51.80 ±23.05_b_^BC^
IVIA2626.1	*pruni*	75.38 ± 15.99_c_^F^	0_a_^A^	59.80 ± 25.27_c_^CDE^	32.26 ± 26.27_b_^ABC^

^α^ Mean ± SD of the percentage of symptomatic leaf area 28 dpi using a cotton swab damped with bacterial inoculum.

Different capital letters indicate statistically significant differences among the assayed strains on each host. Non capital letters indicate significant differences of each strain on four assayed hosts (*p* = 0.05).

The secretory pathway components, and plant cell wall-degrading enzymes, were also analyzed in *X*. *arboricola* genomes (S4 Table). Regarding the encoding elements for the two type II secretory systems (T2SS) described in *Xanthomonas* [31,32], all strains harbored the 11 genes of the *xps* cluster but, in the case of the *xcs* cluster, only CDS homologous to *xcsD*, *xcsE*, *xcsF*, *xcsG* and *xcsJ* were found. The remaining seven components of this secretory system were automatically annotated, but amino acid sequence analysis showed for all of them an identity lower than 80% when compared with the *xcs* cluster described in *X*. *campestris* pv. *campestris* [32].

Variability in the profile of type II secreted virulence factors was found in *X*. *arboricola* (S4 Table). A total of 11 pectolytic enzymes [13,33] were found in this species and only the polygalacturonase, encoded by the locus XCC3459, and the rhamnogalacturonan acetylesterase, encoded by XCC0154 in *X*. *campestris*, were shared by all the strains. With respect to the strains isolated from *Prunus*, they shared, in addition, one pectate lyase (XCC2815), one polygalacturonase (XCC2266) and one rhamnogalacturonase (XAC3505). CITA 44 had homologous sequences to a pectate lyase (XCC0112), a pectin methylesterase (XCC0121) and a pectinmethylesterase (XCC2265) that were absent in strains CITA 33 and IVIA 2626.1, meanwhile these two pathogenic strains had a homologous sequence to the degenerated pectate lyase of *X*. *citri* (XAC2373) which was absent in CITA 44 (Fig 5).

Variation in the profile of cellulolytic enzymes [13] was also found in *X*. *arboricola*, 14 of these degrading molecules were present in this species but only nine of them were shared by all the strains. None of the enzymes were unique in those strains isolated from *Prunus* spp. Pathogenic strains differed from CITA 44 in the presence of a homologous CDS to a beta-glucosidase (XCC1775), and in the absence of the cellulases encoded by the loci XAC3516 and XCC2387, which were present only in CITA 44 (Fig 5, S4 Table).

Moreover, 11 CDS homologous to hemicellulolytic enzymes, previously reported for *Xanthomonas* [13], were found in *X*. *arboricola* and eight of these were shared by all the strains (S4 Table). CITA 33 and IVIA 2626.1 presented a xylosidase which was absent in CITA 44 (Fig 5, S4 Table). Those strains of *X*. *arboricola* which belongs to the pathovars *corylina*, *juglandis* and *pruni* harbored a xylosidase/arabinosidase enzyme which was absent in all the non-pathogenic strains of *X*. *arboricola* and in those strains with a lower pathogenic ability, such as the one described from the pathovar *celebensis*. Finally, the lipase virulence factor, LipA [34], was found in all the genomes analyzed. The *gum* gene cluster, associated with the biosynthesis of xanthan in *Xanthomonas* [35], was analyzed. None of the strains presented homologous sequences to *gumG*, and CITA 44 and 3004 did not show homologous sequences to the *gumF*. (S4 Table).

Other crucial pathogenic elements analyzed were the T3SS components and the type III effectors [13,33,36–42] in the nine genome sequences of *X*. *arboricola* (S4 Table). All the strains harbored CDS that encoded for HpaR2, HpaS, HrpG and HrpX, which are involved in the regulation of the T3SS, and for many of the T3Es and some cell-wall degrading enzymes [43]. However, the strains 3004, CFBP 7634 and CITA 44, as described before [7,17,19], did not present the components of the T3SS that have been found in other *Xanthomonas*. Besides the master regulons mentioned above, all the remaining *X*. *arboricola* strains encompassed and shared 14 hypersensitive response and pathogenicity genes (*hpa2*, *hpaB*, *hrcC*, *hrcJ*, *hrcN*, *hrcR*, *hrcS*, *hrcT*, *hrcU*, *hrcV*, *hrpB1*, *hrpB2*, *hrpB5*, *hrpD6*) (S4 Table).

**Fig 8:**
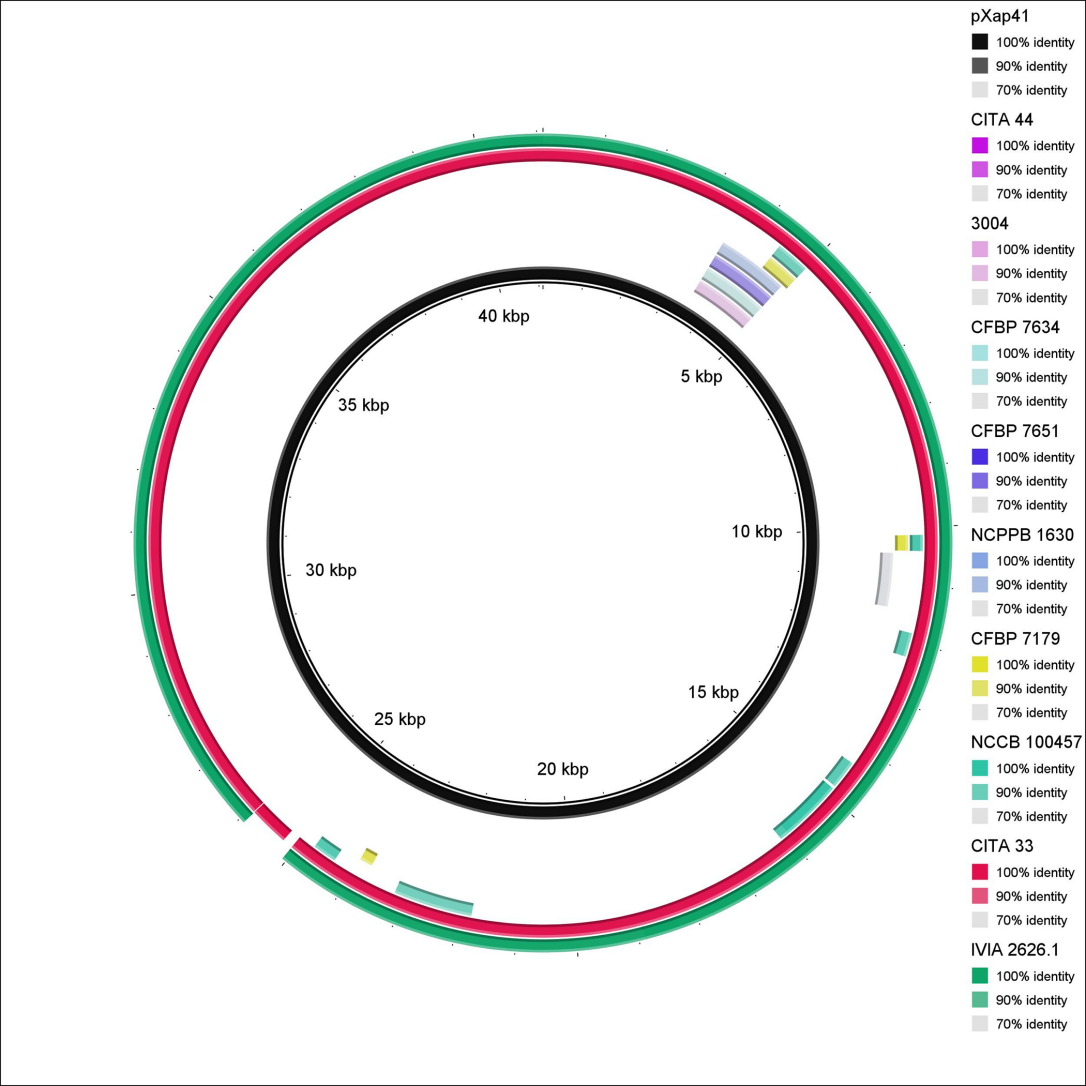
Nucleotide sequence similarity among the sequence of the exclusive plasmid of *X*. *arboricola* pv. *pruni* pXap41 and the draft genome sequences of nine strains of *X*. *arboricola*. Blastn was used for the comparative sequence analysis with an expected value threshold of 0.001. Circular graphic representation was constructed using the BLAST Ring Image Generator (BRIG) tool. See legend for information related to each ring of the map.

The T3E profile of *X*. *arboricola* composed of 22 virulence effectors (*avrBs2*, *avrXccA2*, *hpaA*, *hrpW*, *xopA*, *xopAF*, *xopAH*, *xopAI*, *xopAQ*, *xopB*, *xopE2*, *xopE3*, *xopF1*, *xopG*, *xopK*, *xopL*, *xopN*, *xopQ*, *xopR*, *xopV*, *xopX* and *xopZ1*). The non-pathogenic strain CITA 44 from *Prunus*, and the pathogen of barley strain 3004, did not have any T3Es. The remaining non-pathogenic strains, CFBP 7634 and CFBP 7651, harbored two (*avrsB2* and *xopR*) and six (*avrsB2*, *hpaA*, *hrpW*, *xopA*, *xopF1* and *xopR*) T3Es, respectively [7] (S4 Table). In addition, the pathogenic strain NCPPB 1630 from *Musa* sp. showed six T3Es, the pathogenic strain CFBP 7179 from *J*. *regia* showed 16 T3Es and the strain NCCB 100457, that causes disease on *C*. *colurna*, showed 19 T3Es. *X*. *arboricola* pv. *pruni* strains CITA 33 from *P*. *amygdalus* and IVIA 2626.1 from *P*. *salicina* harbored 21 T3Es. All the strains, regardless of their hosts, shared six T3Es (*avrBs2*, *hpaA*, *hrpW*, *xopA*, *xopF1* and *xopR*). The effector *xopB* was unique in the strain that causes disease on *Juglans*, meanwhile *xopAQ*, which has not been previously reported in *X*. *arboricola*, and *xopE3*, were only found in those strains that cause disease on *Prunus*. CDS homologous to these two T3Es were specifically present in the plasmid pXap41, which is unique in the strains of the pathovar *pruni* [44] (Fig 8).

## Discussion

MLSA has been shown to be useful to characterize strains of *X*. *arboricola* [2,5,18,45,46]. In this work, we utilized a MLSA scheme proposed by Young and collaborators [18], which is based in the analysis of partial sequences of the genes *dnaK*, *fyuA*, *gyrB* and *rpoD*. Here, we have concluded that for 14 *X*. *arboricola* strains isolated from *Prunus*, the selected MLSA scheme was a good approach to characterize and discriminate the members of the pathovar *pruni* from atypical or commensal strains of *X*. *arboricola*.

Classification of CITA 44 as an atypical strain of *X*. *arboricola* was corroborated by the phylogenetic analysis conducted with 2,525 genes identified as the components of the core genome of this species. This atypical strain isolated from *Prunus* did not present the same phylogenetic origin as the pathogenic strains classified as pathovar *pruni*, but it was more similar to strain 3004 of *X*. *arboricola*, which did not cluster within any of the well-established pathovars of this taxon. Even though CITA 44 was closely related to 3004, this strain did not produce symptoms on inoculated barley in assays performed in our group. This result is similar to a previous work on *X*. *arboricola* strains isolated from walnut that were more similar to strains isolated from *Musa* sp. than to those of the pathovar *juglandis* isolated from walnut [7].

Moreover, the study of these atypical strains, found on barley, walnut and here in *P*. *mahaleb*, open the discussion about the origin and evolution of pathogenicity in this species as it has been proposed previously for the genus *Xanthomonas* [43]. Based in the current data, it is not possible to determine if these non-pathogenic strains were predecessors of the pathogenic groups or the result of the loss of their pathogenicity. Further exploratory studies regarding some other *Prunus*-associated strains with other phenotypic and genotypic variants are needed to elucidate the evolutionary process of pathogenicity in this bacterial species.

In addition, this study contributes to our understanding of the diversity of *X*. *arboricola* associated with stone fruit trees and almond. Moreover, it also provides information to develop tools to identify and discriminate pathogenic and non-pathogenic *Xanthomonas* strains found in *Prunus* spp. This precise characterization of atypical strains of *X*. *arboricola* will avoid bacterial identification mistakes, as occurred sometimes in other bacterial models which implied unnecessary control measures and resulted in high economic losses [47,48].

The phylogenetic variation among strains of *X*. *arboricola* isolated from *Prunus* concurred with the existence of differences in several phenotypic features including pathogenicity. Additionally, genomic information generated from xanthomonads isolated from *Prunus* [16,17] and from other *X*. *arboricola* [7,19–23], has been used to reveal those features which could be involved in the disease process.

Carbon source utilization profiles, as expected [1,2,5], showed high homogeneity among strains of the pathovar *pruni*. Nevertheless, the profile of carbon sources utilization shown by CITA 44 was different to the one presented by the other strains as well as the one provided in the description of *X*. *arboricola* species [1]. This disparity from the original metabolic description of the species has been also described for some strains of other *Xanthomonas* species, such as *X*. *vesicatoria* [49] and *X*. *campestris* pv. *campestris* [50]. The discordant profile of CITA 44 could be due to the fact that the original description of the species *X*. *arboricola* was based on strains of seven pathogenic pathovars which presented a high intra-pathovar homogeneity [1,2] and did not consider a wider ecological diversity including the atypical non-pathogenic strains currently of great research interest.

Initial stages of bacterial adaptation and host colonization encompass a series of sensors and receptors that detect stimuli, providing to the cells the information of their biotic and abiotic environment which triggers a series of processes such as the cell motility, chemotaxis, quorum sensing, biofilm formation and many other cellular events [15,51]. At this stage, a group of protein complexes denominated TBDTs, STCRS and MCPs play a crucial role [25].

The repertoire of TBDTs in *X*. *arboricola*, which are bacterial outer membrane proteins associated with the transport of different substrates including carbohydrates [52], was extensive compared to other species of this genus [25,33], resulting similar to the one observed in *X*. *campestris* and other epiphytic xanthomonads [25]. Additionally, this repertoire is in accordance with the one previously observed in two not publicly available genome sequences of *X*. *arboricola* pv. *fragariae* (strains LMG 19145 and LMG 19146) and one of *X*. *arboricola* pv. *pruni* (strain LMG 25862) [33]. This high number of TBDTs has been associated in other xanthomonads with the need for carbohydrates scavenging in variable conditions encountered in epiphytic niches [53].

Bacterial sensing and chemotaxis are essential components of the initial infection processes [54]. Regarding STCRS proteins, which are part of the dominant molecular mechanism by which unicellular organisms respond to environmental stimuli, the large repertoire observed in *Xanthomonas arboricola* was similar to that observed in *X*. *campestris* [25], and it was in accordance with the number of STCRS observed in most of the complete genome sequences of *Xanthomonas*, with the exception of those species which inhabit in restricted niches such as *X*. *albilineans* [15,51]. Evaluation of TBDTs and STCRS content revealed differences between CITA 44 and *X*. *arboricola* pv. *pruni* strains, despite of the general low variability observed among the different *X*. *arboricola* strains evaluated.

Beside this, CITA 44, compared to strains of the pathovar *pruni*, also showed a distinct chemotactic pattern, being influenced by just a few compounds. This kind of intraspecific chemotactic variability has been also observed in other xanthomonads models like *X*. *citri* subsp. *citri* with dissimilar pathogenic ability [24].

In order to explain these chemotactic behaviors, MCP content was evaluated. MCPs sense beneficial or toxic environmental compounds and transduce the signal to the cytoplasm by CheW protein, causing changes in the flagella direction and rotation speed, which finally guides bacterial cells to favorable environments [54,55]. Here, we have found that the core repertoire of the MCPs in nine genome sequences of *X*. *arboricola* included at least 13 chemoreceptors. Differences in the MCPs content were observed among the genome-sequenced strains and could be associated with their host range. Differences in MCPs content were found between CITA 44 and other xanthomonads from *Prunus* spp. Moreover, a variation in the *cheW* locus was also identified.

Although functional analyses are necessary to confirm the role of these sensors at initial stages of the infection process, our results point out that these processes and the genes involved may mark the diverse behavior of the different strains. Moreover, the very initial stages of the bacterial-host interaction described above could trigger other molecular routes which involve components such as the flagella and the type IV pilus, that are related not only to motility on liquid or solid environments, but also with attachment to the host or the development of biofilm structures [30,56,57]. Motility on solid and semisolid surfaces, which are controlled by flagella or type IV pilus, was also variable among the assayed strains of *X*. *arboricola* isolated from *Prunus*. This variability among different strains in swimming, swarming and twitching motility have been shown in other xanthomonads such as *X*. *citri* [58], as well as in *X*. *arboricola* strains isolated from walnut [7]. Strain CITA 44 showed the higher ability to swim that maybe connected to a more restricted niche to survive and higher requirements to locate it. Similarly, this enhanced ability to swim has been described in other bacterial models like *X*. *citri* subsp. *citri*, which less virulent strains were described to have a higher swimming ability [58]. In relation to the surface motility in *X*. *arboricola*, two different colony phenotypes were observed; the circular one matched with the one observed for other species such as *X*. *oryzae* and *X*. *citri* [59,60], described previously as independent of flagella and defined as sliding type motility instead of swarming [57]. Contrary to this, some other strains showed dendritic swarmer colonies, which had not been previously described in *Xanthomonas* but confirmed in other bacterial species such as *Pseudomonas aeruginosa* as a real swarming type motility [61]. In addition, these strains showed other swarming-related features such as the production of surfactants and a rapid outward migration [62]. Again, CITA 44 showed a different pattern to that of *X*. *arboricola* pv. *pruni*, presenting a colony with an intermediate phenotype between dendritic and circular and also an intermediate surfactant production.

Genome analysis of the flagellum components in CITA 44 revealed an interesting amino acid substitution in the N-terminal region of the flagellin that was also found in other non-pathogenic xanthomonads. Previously, Cesbron and collaborators [7] reported an amino acid polymorphism (Asp-43/Val-43) in the flagellin domain flg22 among pathogenic and non-pathogenic strains of *X*. *arboricola* isolated from walnut. Here we report that this variation is not only present in *X*. *arboricola* pv. *juglandis* strains but it is also found in the other two major pathogenic pathovars of this species, *corylina* and *pruni*. In *X*. *campestris*, strains with Val-43 in flg22 were not detected by the flagellin sensing 2 kinase (FLS2) of *Arabidopsis*, therefore they did not elicit the pathogen-associated molecular (PAMP)-triggered immunity and, additionally, these strains were more virulent than those with an Asp-43 residue in flg22 [63]. The genomic analysis also revealed the absence of *fliD* in CITA 44. In several bacterial species the absence of *fliD* was associated with non-flagellated and non-motile cells [64,65]. This is not the case of CITA 44, which showed a single polar flagellum like *X*. *arboricola* pv. *pruni* strains [17].

The type IV pilus is an important structure related to the movement across the surface, adhesion, microcolony formation, secretion of proteases and colonization factors, being a key pathogenesis factor [66]. In general, strains isolated from *Prunus*, despite their pathogenicity, harbor all the four subcomplexes that permit the biogenesis and function of this structure [30]; additionally these components were demonstrated as functional in the twitching motility assay.

As the type IV pilus, non-fimbrial adhesins are involved in bacterial attachment to host surfaces; in *X*. *oryzae*, they play an important role in pathogenesis and are particularly involved at the initial stage of leaf attachment and penetration into the host [67,68]. *X*. *arboricola*, shows various combinations of adhesins among the different strains studied. This variability could be associated to the different bacterial-plant compatible interactions of the different strains and hosts [69]. Once more, CITA 44 lacked one of the genes involved in adhesion synthesis which was present in *X*. *arboricola* pv. *pruni*. Further functional studies in this way could be valuable to define the definitive role of these adhesins in the pathogenicity and host specificity observed in the pathovars of *X*. *arboricola*.

After the very initial stages of pathogenesis described above, some virulence genes related to host colonization, multiplication and development of symptoms are expressed. T2SS permits the export of proteins from the bacterial cell and it is involved in the translocation of degradative enzymes which causes damage to the host cells and tissues [70]. Most species of *Xanthomonas* encode the components of two T2SS. One of them is Xps, which contributes to bacterial virulence by the secretion of xylanases and proteases in *X*. *campestris* and *X*. *oryzae* [32]. In *X*. *arboricola*, all the components of the *xps* gene cluster were conserved. As in all the *Xanthomonas* species, *X*. *arboricola* showed a core repertoire of cell wall degrading enzymes which is formed by at least 30 CDS. Beside this, a high variation in the repertoire of these enzymes was also found among the analyzed strains. These variations could be related to the different cell wall composition of the hosts or tissues and the requirements to produce symptoms during the infection [69]. Differences in the profile of degrading enzymes were identified among the *Prunus*-pathogenic strains and other strains of *X*. *arboricola*, including CITA 44.

Another gene cluster associated with pathogenesis in *Xanthomonas* is the xanthan producing *gum* gene cluster which is composed by 12 genes. Deletion of this group of genes inhibits the disease development in *X*. *campestris* on *Arabidopsis* and *Nicotiana* [71]. In *X*. *arboricola*, this cluster was conserved, with the exception of *gumG* that was absent in all the sequenced strains and the O-acetyltransferases encoded by *gumF* lacking in strains CITA 44 and 3004. Nevertheless, the absence of these two genes has been demonstrated to not affect the xanthan polymerization [72].

T3SS, as well as the T3Es, plays an essential role in pathogenicity once bacteria have penetrated the host tissue and could be related to host specificity in *Xanthomonas* [37]. As occurred in other *Xanthomonas* species, all the *X*. *arboricola* strains harbored orthologs CDS to the genes that encode HpaR2, HpaS, HrpG and HrpX, which are involved in the regulation of T3SS, T3Es as well as other pathogenic factors such as those encoded by *pehA* and *pehD* genes [43]. This was also previously reported in the strains isolated from walnut as well as for strains 3004, CITA 44 and IVIA 2626.1 [7,17,43].

It is important to note the localization in *X*. *arboricola* of an ortholog to the T3E, called *xopAQ*, in the plasmid pXap41, next to other two virulence associated proteins, *xopE3* and *mltB* [44]. This effector has been previously found in the chromosome of *X*. *citri* and *X*. *gardneri* and a similar sequence has been found in *Ralstonia solanacearum* [40,73]. Nucleotide sequence analysis revealed a high pairwise nucleotide identity (99.7%) to the *xopAQ* sequence of *X*. *citri* subsp. *citri* A^w^12879, as well as a putative plant-inducible promoter box (PIP-box) sequence 67 bps upstream the start codon, but this conserved sequence associated with the regulon HrpX showed a variant in one nucleotide (S4 Fig). The presence of at least three T3Es in pXap41 reinforces the hypothesis that this plasmid may contribute to the virulence of this pathovar as well as to the specialization of the pathovar *pruni* towards hosts of the *Prunus* genus [44]. CITA 44 did not show *xopAQ* or any of the T3Es found, which is consistent with the absence of pXap41 and its non-pathogenic character. Currently, functional studies on the effect of pXap41 and its related T3Es are being conducted in our group to confirm their role in the pathogenicity and the host specificity of the pathovar *pruni* strains.

In conclusion, this study of strains with diverse virulence range, and particularly strain CITA 44, has provided an opportunity to elucidate essential mechanisms in the host-bacteria interaction in *Xanthomonas arboricola* pv. *pruni*. In addition, it is useful to understand the evolution of the pathogenicity in this species. Furthermore, the comparative analysis reported here highlights several differences regarding to key genotypic and phenotypic features of initial and late stages of the infection process. The different TBDT, STCR, MCP profiles, the genomic variation in the components of flagellin, the type IV pilus and in cell-wall degrading enzymes repertoire, and the alteration in main virulence factors provide information that contributes to explain the phenotypic differences observed between pathogenic and non-pathogenic strains. Although the work presented shows a global overview of mechanisms involved in virulence, further functional studies are needed to test and improve the understanding of the role of all the virulence-related features mentioned here.

## Materials and Methods

### Bacterial strains and culture conditions

*X*. *arboricola* strain CITA 44, isolated from asymptomatic leaves of *P*. *mahaleb* in the Spanish region of Aragón, and fourteen bacterial strains of *X*. *arboricola* pv. *pruni* isolated from trees showing bacterial spot symptoms in Spain and Italy, were used in this study (Table 1). From these fourteen strains, three were isolated from almond [*Prunus amygdalus* Batsch, syn. *P*. *dulcis* (Miller) D. A. Webb], four from peach [*Prunus persica* (L.) Batsch], six from Japanese plum (*Prunus salicina* Lindley) and one from the hybrid rootstock Garnem (GxN15) (*P*. *persica* x *P*. *amygdalus*). Reference strains of *X*. *arboricola* pv. *pruni* (10.0343, 10.0400, 10.439, CFBP 3894, CFBP 5530, CFBP 5724), *X*. *arboricola* pv. *corylina* (CFBP 1846), *X*. *arboricola* pv. *populi* (CFBP 3123) and *X*. *arboricola* pv. *juglandis* (IVIA 2113) were also included in this study (Table 1). Strains were routinely cultured on 1.5% agar plates of Luria Bertani (LB) or in LB broth at 27°C for 48 h. All *Xanthomonas* strains are conserved in the collections of Centro de Investigación y Tecnología Agroalimentaria de Aragón (CITA, Aragón, Spain) and Instituto Valenciano de Investigaciones Agrarias (IVIA, Valencia, Spain) and no specific permission was required for their use.

### Multi Locus Sequence Analysis (MLSA)

Bacterial DNA was extracted from cultures in LB broth obtained after 24 h incubation at 27°C, using a QIAamp DNA miniKit according to the manufacturer's instructions (Qiagen, Barcelona, Spain). DNA was used for PCR or stored at -20°C until further use. Degenerate primers, previously determined as useful in MLSA analysis conducted in *Xanthomonas* [18,46], were used for PCR amplification of partial sequences of the housekeeping genes *dnaK*, *fyuA*, *gyrB* and *rpoD*. PCR amplifications were carried out in a 50 μL volume containing 1X PCR buffer (10 mM Tris-HCl, 50 mM KCl, 0.1% Triton X-100 [pH 9.0]); 0.2 μM of each primer; 1.25 U *Taq* DNA polymerase (Biotools, Madrid, Spain); 0.2 mM each dNTP (Biotools Madrid, Spain); 1.5 mM MgCl_2_ and 1.0 μg/μL of DNA template. All PCR reactions were performed in an ABI 2720 thermal cycler (Applied Biosystems, Foster Urban district, CA, USA) with an initial denaturation at 94°C for 5 min, 40 cycles of denaturation at 94°C for 1 min, annealing at 55°C for 1 min and extension at 72°C for 2 min, and a final extension step at 72°C for 10 min. PCR products were visualized under UV light in 2% agarose gels stained with ethidium bromide and purified with the Wizard SV Gel and PCR Clean-up System Kit (Promega Corporation, Madison, USA). PCR products were sequenced at STAB VIDA (Lisbon, Portugal), and edited using BioEdit Sequence Alignment Editor [74]. Additionally, sequences of the housekeeping genes used for MLSA analysis from *X*. *arboricola* pathovars *celebensis* (ICMP 1488), *corylina* (ICMP 5726) and *juglandis* (ICMP 35) as well as *X*. *axonopodis* pv. *citri* strain ICMP 24, included as outgroup, were obtained from the National Center for Biotechnology Information database (NCBI) (http://www.ncbi.nlm.nih.gov).

Nucleotide sequences were aligned with ClustalW version 1.83 [75] using default parameters. Both ends of each alignment were trimmed to the following sizes: *dnaK*, 864 positions; *fyuA*, 607 positions; *gyrB*, 631 positions and *rpoD*, 756 positions. Nucleotide sequences from all strains analyzed here and from those *Xanthomonas* spp. available in databases [18] were aligned and concatenated to give a total length of 2,858 nucleotide positions. The programs jModelTest 0.1.1 [76] and MEGA 5.05 [77] were used to determine the best model of evolution for Maximum Likelihood analysis (ML) based in the akaike information criterion (AIC) [78]. For the concatenated gene dataset the model selected was TN93+G. Maximum likelihood trees, using 1,000 bootstrap re-samplings, were generated in MEGA 5.05.

Nucleotide sequences were deposited in GenBank. Accession numbers for the partial sequences of the genes used in this study are: KR054426 to KR054449 for *dnaK*; KR054450 to KR054473 for *fyuA*; KR054474 to KR054497 for *gyrB* and KR054498 to KR054521 for *rpoD*.

### Genomic Analysis

Based in the MLSA analysis, three *X*. *arboricola* strains, CITA 33 (Synonymy Xap 33), CITA 44 and IVIA 2626.1, were selected for complete genome sequencing and genome features for these strains have been previously announced [16,17]. Nucleotide sequences of the draft genome sequences of these three strains (Deposited at DDBJ, EMBL, GenBank under the accession numbers JHUQ00000000 for CITA 33, LJGM00000000 for CITA 44, and LJGN00000000 for IVIA 2626.1), as well as nucleotide sequences of three strains of *X*. *arboricola* (3004, CFBP 7634 and CFBP 7651) [7,19], two strains of *X*. *arboricola* pv. *celebensis* (NCPPB 1630 and NCPPB 1832) [23], one strain of *X*. *arboricola* pv. c*orylina* (NCCB 100457) [20], eight strains of *X*. *arboricola* pv. *juglandis* (CFBP 2528, CFBP7179, NCPPB 1447, XajA3, Xaj2, Xaj4-1, Xaj43a and Xaj417) [7,21,22] and one strain of *X*. *arboricola* pv. *pruni* (MAFF 301420) were obtained from the NCBI´s genome database (S1 Table). Genome scaffolds presented in each one of the bacterial genomes mentioned above were arranged and oriented by Mauve [26] using the complete genome sequence of *X*. *arboricola* pv. *juglandis* strain Xaj417 [22] as reference.

For the purpose of homogeneity for further comparison, all the genome sequences were annotated using Prokka [79]. GFF3 archives generated by Prokka were used as the input to determine the core and the dispensable accessory genes in the analyzed genomes using Roary [80]. The online tool available at bioinformatics.psb.ugent.be, was used to generate the Venn diagram to compare the genome composition of the *Prunus-*isolated strains with the remaining subspecific groups of *X*. *arboricola*. Signal peptides and transmembrane domains for the unique protein coding sequences (CDS) of *X*. *arboricola* CITA 44 and *X*. *arboricola* pv. *pruni* CITA 33 or IVIA 2626.1, were determined using the signalP 4.1 server and the TMHMM server version 2.0 [81,82]. Beside this, the assignment of these genes to the clusters of orthologous groups (COG) database [83] was performed with the NCBI`s conserved domain database using an expected value of 0.001 [84].

The core genome sequence obtained for each strain was aligned using MAFFT [85] for further phylogenetic analysis. Subsequently, a maximum likelihood tree, using 1,000 bootstrap resamplings, was constructed to accurately determine the phylogenetic position of the atypical strain isolated from *Prunus*, CITA 44, within *X*. *arboricola*. Maximum likelihood tree was carried out using the RaxML tool [86] and the dendrogram obtained was visualized using Dendroscope [87]. *X*. *campestris* pv. *campestris* strain ATCC 33913 was used as an outgroup in the analysis.

### Carbon sources utilization analysis and environmental sensors profile

To prepare the inocula, bacterial strains used in the MLSA analysis (Table 1) were cultured in LB plates and resuspended in sterile phosphate-buffered saline (PBS) (OD_600_ = 0.3). Suspensions (150 μL) were inoculated into each well of the Biolog GN2 microplates (Biolog Inc., USA). Microplates were read at 570 nm using a Labsystems Multiskan RC spectrophotometer (Fisher Scientific, Walthman, USA) after 24 h incubation at 27°C [1,88]. Three independent assays were performed, each including two microplates per strain and three reads per well. Means from all the reads were performed and used in further analysis.

Three substrate utilization levels were defined based in the percentage of utilization referred to water control [89]: positive (+, > 160%); borderline, considered as non-informative (±, 130–160%) and negative (-, < 130%).

The carbon sources utilization profile of strain CITA 44 was determined and compared with those obtained for strains of the pathovars *corylina*, *juglandis*, *populi* and *pruni*; results were converted to a binary form by scoring the metabolism observed for each compound as 0 (no catabolism of the carbon source) and 1 (catabolism of the carbon source). Similarity for pairs was calculated according to the Jaccard´s coefficient and was subjected to Unweight Pair Group Method with Arithmetic Mean (UPGMA) cluster analysis. Finally, the reliability of the similarity trees was determined using the cophenetic correlation index. All the analysis were computed on NTSYS 2.11T (Exeter Software, Setauket, NY).

Profiles of sensors of two-component regulatory system (STCRS) and TonB-dependent transporters (TBDTs) for *X*. *arboricola* strains isolated from *Prunus* (CITA 33, CITA 44 and IVIA 2626.1) and six other *Xanthomonas* (3004, CFBP 7634, CFBP 7651, NCPPB 1630, NCCB 100457 and CFBP 7179) (S1 Table) were determined based in the analysis of the complete genomes and the search of homologous sequences for 86 STCRS and 28 TBDTs previously described in other *Xanthomonas* species [25]. Sequence homology searches were conducted using the Blast tool according to the protocol proposed by the NCBI [90], using the nucleotide sequence that encodes each one as the input. Those CDS which presented an identity and a query coverage percentage over 80% were considered as orthologous genes in the target genomes of *X*. *arboricola*.

### Chemotaxis assay and repertoire of methyl accepting chemotaxis proteins

Eighteen carbon compounds that induce variation in the chemotactic response in other *Xanthomonas* [24] were evaluated to determine differences in the chemotactic profile between the atypical strain CITA 44 and *X*. *arboricola* pv. *pruni* strains CITA 33 and CFBP 5530, for which complete genome sequence had been already obtained [16,17,91]. The chemotactic compounds tested were: alanine (10 and 250 mM), arginine (10 and 100 mM), citric acid (10 mM), cysteine (10 mM), fructose (10 mM), galactose (10 mM), galacturonic acid (10 mM), glucose (10 mM), glucuronic acid (10 mM), glycerol (0.2 and 2%), leucine (10 and 150 mM), maltose (10 mM), mannitol (0.2%), serine (10 and 200 mM), sodium citrate (10 mM), succinic acid (10 mM), sucrose (10 mM) and xylose (10 mM).

The chemotactic effect of the carbon compounds was evaluated according to a microtiter plate chemotaxis assay previously developed [24]. Briefly, 10 μL tips containing 5 μL of the carbon source tested were inserted into 48 of a 96 wells plate previously inoculated with 200 μL of bacterial suspension in 10 mM MgCl_2_ (10^8^ CFU/mL). The number of bacteria that moved into the tip during one hour was estimated by means of serial dilutions of the tip content plated on 1.5% agar LB plates. Significant differences (*p* < 0.05) between the means from each carbon source tested and the negative control (10 mM MgCl_2_) were determined by an analysis of variance (ANOVA) according to the Student-Newman-Keuls method. Statistical analyses were performed using STATGRAPHICS Plus v.5.1 (Manugistics Inc. Rockville Maryland, USA). A carbon source was considered as a chemoattractant when the average number of bacteria contained in the tip (6 replicates in 2 independent assays) was significantly higher to the blank control (10 mM MgCl_2_), and considered as a chemorepellent when the average was significantly lower (*p* < 0.05).

Orthologous genes for the 26 methyl accepting chemotaxis proteins (MCPs) as well as for 18 specific-chemotaxis *che* genes [25], described in *X*. *campestris* pv. *campestris*, *X*. *campestris* pv. *vesicatoria*, *X*. *citri* subsp. *citri* and *X*. *oryzae*, were searched in the genome sequences of nine *X*. *arboricola* strains as mentioned above. Those CDS with more than 80% of identity and more than 80% of sequence coverage containing MCPs periplasmic domains were considered as orthologous genes.

### Motility in solid and semisolid surfaces and surfactant activity

Swarming, swimming and twitching motility assays were conducted for strain CITA 44 as well as for 20 strains classified as *X*. *arboricola* pv. *pruni* by MLSA. Bacterial cultures in exponential growth phase were centrifuged at 6,350 *g* for 15 min, then washed and resuspended in 10 mM MgCl_2_ (OD_600_ = 1.0). To analyze swarming motility, 0.5% agar PYM swarming plates (peptone 0.5%, yeast extract 0.3%, malt extract 0.3%, glucose 1.0%) were inoculated with 10 μL of bacterial suspension. For swimming motility assay, cultures resuspended in 10 mM MgCl_2_ were centrifuged at 6,350 *g* during 15 min and the pellets were inoculated using a sterile toothpick in the center of semisolid 0.3% agar minimal medium A (MMA) plates (K_2_HPO_4_ 0.7%, KH_2_PO_4_ 0.3%, MgSO_4_·7H_2_O 0.01%, (NH_4_)_2_SO_4_ 0.1%, sodium citrate 0.005%, glycerol 0.2%). For twitching assay, bacterial cultures were prepared as mentioned above, and then inoculated with a sterile toothpick through a 5 mm PYM 1.5% agar layer to the bottom of the plate. After 72 h, culture medium was removed and the bottom of the plate was stained with 0.3% crystal violet for 15 min and washed using sterile distilled water.

Surfactant production, which is closely related to bacterial motility on solid surfaces, was also assessed according to the atomized oil assay [27]. Briefly, bacteria were inoculated using a sterile toothpick on 1.5% agar LB plates, and after 24 hpi, a fine mist of mineral oil droplets were sprayed on the colony. Bacterial strains that instantaneously showed a bright halo around the colony were recorded as surfactant producers.

Plates from swarming motility and surfactant activity assays were incubated at 27°C during 24 h, whilst swimming and twitching plates were incubated for 72 h. Images from all the plates were recorded. All assays were performed in three independent experiments with three replicates each time.

Homologous CDS to the major structural components associated with flagellum [25,29,92] as well as the fimbrial adhesin type IV pilus [30] and a group of non-fimbrial adhesins [25] were identified in the three genomes of *X*. *arboricola* isolated from *Prunus* and in other six *X*. *arboricola* strains as described above.

### Pathogenicity tests on *Prunus* spp

Bacteria were grown on 1.5% agar LB plates and incubated at 27°C for 48 h, then a single bacterial colony was resuspended in 30 mL of LB broth and incubated at 27°C on a rotary shaker for 24 h. After incubation, cultures were centrifuged at 6,350 *g* for 15 min and washed three times with 10 mM MgCl_2_. Finally, cultures were adjusted to 10^8^ CFU/mL (OD_600_ = 0.1) and used to inoculate detached leaves of different *Prunus* species.

Young, fully expanded leaves from greenhouse-grown plants of almond (cv. Ferraduel), apricot (cv. Canino), peach (cv. Calanda) and European plum (cv. Golden Japan) were collected, brought to the laboratory and washed three times in sterile distilled water. After surface sterilization with 70% ethanol and 0.05% sodium hypochlorite, the leaves were rinsed three times with sterile distilled water and dried on a hood. Three leaves per host were selected randomly for being inoculated with each strain in three independent assays.

Surface sterilized leaves were placed on 0.5% water agar plates and the abaxial surfaces were gently inoculated using a sterile cotton swab damped with the bacterial inoculum. Sealed plates were incubated in a grow chamber adjusted at 27°C, 80–90% relative humidity and 12 h photoperiod. Inoculated leaves were digitally recorded at 28 dpi and the percentage of symptomatic area per host and strain was quantified using the ImageJ 1.45s software. Samples, inoculated with sterile 10 mM MgCl_2_, were used as blank control for comparison.

Additionally to the genomic analysis related to the early events in the bacteria-host interaction described below, homologous CDS to several genes associated with later stages of pathogenesis, like the type III secretory system (T3SS) and the type III effectors (T3Es), were searched in the genome of the nine strains of *X*. *arboricola* according to the percentage of the sequence length and identity with the amino acid sequence of the genes previously associated with these features [36–41,93,94]. In the same manner, orthologous CDS to other remarkable processes and features associated with virulence such as the quorum sensing [95], the xanthan biosynthesis [35], the type II secretion system [31,32] as well as the cellulolytic, hemicellulolytic, pectolytic and lipases enzymes, previously described in other species of *Xanthomonas* [13,33,34,96], were searched in the genome sequences of the nine strains of *X*. *arboricola*. Only those CDS with a percentage of coverage and identity over 80.0% were considered as orthologous sequences.

Finally, the presence of the plasmid pXap41 [44], putatively involved in virulence in *X*. *arboricola* pv. *pruni*, was searched in the analyzed genomes based in the nucleotide sequence similarity and graphically represented using the BLAST Ring Image Generator (BRIG) tool [97]; blastn was used for the sequence comparative analysis with an expected value threshold of 0.001. All the CDS associated with pathogenesis, mentioned above, were represented in a circular genome map using CGView [98]. For this purpose, contigs of the draft genome sequence of CITA 33, CITA 44 and IVIA 2626.1 were arranged by Mauve [26] using the circularized genome sequence of *X*. *arboricola* pv. *juglandis* strain Xaj 417 as the reference [22].

## Supporting Information

S1 Fig Nucleotide genome sequence comparison of *X*. *arboricola* pv. *pruni* strains CITA 33 and IVIA 2626.1 and the non-pathogenic strain CITA 44. Genome sequences were compared against each other based on Blastn results and represented as a circular map using the CGview tool. From outside to center: strain CITA 33, strain IVIA 2626.1, strain CITA 44, GC content, GC skew + and GC skew -.
(TIF)

S2 Fig Transmission electron microscopy of *X*. *arboriola* strain CITA 44. Negative staining showed monoflagellated cell from the edge of the dendritic swarming colony 24 hpi in 0.5% PYM agar plates.
(TIF)

S3 Fig Amino acid alignment of two variants of the FliC found in *X*. *arboricola*. Sequence alignment, performed using ClustalW, showed the amino acid change in the position number 43 of the amino terminal region of FliC.
(TIF)

S4 Fig Nucleotide alignment of type III effector *xopAQ* and the PIP-box found in *X*. *arboricola* pv. *pruni*. Sequence alignment, performed using ClustalW, showed a slight variation among *X*. *arboricola* and other *Xanthomonas* (A) as well as a variant in PIP-box sequence (B).
(TIF)

S1 Table Genome statistics of 18 *X*. *arboricola* strains according to the automatic annotation performed using Prokka. (XLSX)

S2 Table Annotated protein sequences shared by 18 pathogenic and non-pathogenic strains of *X*. *arboricola*. (XLSX)

S3 Table Unique protein coding sequences (CDS) and related features found in the draft genome sequence of *X*. *arboricola* strain CITA 44 and *X*. *arboricola* pv. *pruni* strains CITA 33 and IVIA 26262.1. (XLSX)

S4 Table Orthologous protein coding sequences (CDS) of nine strains of *X*. *arboricola* associated with pathogenesis. (XLSX)

## References

[1] Vauterin L, Hoste B, Kersters K, Swings J. Reclassification of *Xanthomonas*. Int J Syst Bacteriol. 1995;45 (3): 472–489.

[2] Fischer-Le Saux M, Bonneau S, Essakhi S, Manceau C, Jacques M-A. Aggressive emerging pathovars of *Xanthomonas arboricola* represent widespread epidemic clones distinct from poorly pathogenic strains, as revealed by multilocus sequence typing. Appl Environ Microbiol. 2015;81 (14): 4651–4568. 10.1128/AEM.00050-15 25934623 PMC4551192

[3] EFSA PLH Panel (EFSA Panel on Plant Health). Scientific opinion on pest categorisation of *Xanthomonas arboricola* pv. *pruni* (Smith, 1903). EFSA J. 2014;12: 3857–3882.

[4] Stefani E. Economic significance and control of bacterial spot/canker of stone fruits caused by *Xanthomonas arboricola* pv. *pruni*. J Plant Pathol. 2010;92 (1, Supplement): S1.99–S1.103.

[5] Boudon S, Manceau C, Nottéghem JL. Structure and origin of *Xanthomonas arboricola* pv. *pruni* populations causing bacterial spot of stone fruit trees in Western Europe. Phytopathology. 2005;95 (9): 1081–1088. 10.1094/PHYTO-95-1081 18943306

[6] Denancé N, Lahaye T, Noël LD. Editorial: Genomics and effectomics of the crop killer *Xanthomonas*. Front Plant Sci. 2016;7: 71 10.3389/fpls.2016.00071 26870077 PMC4735375

[7] Cesbron S, Briand M, Essakhi S, Gironde S, Boureau T, Manceau C, et al Comparative genomics of pathogenic and non-pathogenic strains of *Xanthomonas arboricola* unveil molecular and evolutionary events linked to pathoadaptation. Front Plant Sci. 2015;6: 1126 10.3389/fpls.2015.01126 26734033 PMC4686621

[8] Essakhi S, Cesbron S, Fischer-Le Saux M, Bonneau S, Jacques M-A, Manceau C. Phylogenetic and VNTR analysis identified non-pathogenic lineages within *Xanthomonas arboricola* lacking the canonical type three secretion system. Appl Environ Microbiol. 2015;81 (16): 5395–5410. 10.1128/AEM.00835-15 26048944 PMC4510168

[9] Vernière CJ, Gottwald TR, Pruvost O. Disease development and symptom expression of *Xanthomonas axonopodis* pv. *citri* in various citrus plant tissues. Phytopathology. 2003;93 (7): 832–43. 10.1094/PHYTO.2003.93.7.832 18943164

[10] Gordon JL, Lefeuvre P, Escalon A, Barbe V, Cruveiller S, Gagnevin L, et al Comparative genomics of 43 strains of *Xanthomonas citri* pv. *citri* reveals the evolutionary events giving rise to pathotypes with different host ranges. BMC Genomics. 2015;16: 1098 10.1186/s12864-015-2310-x 26699528 PMC4690215

[11] Kamoun S, Kado CI. Phenotypic switching affecting chemotaxis, xanthan production, and virulence in *Xanthomonas campestris*. Appl Environ Microbiol. 1990;56 (12): 3855–3860 16348384 10.1128/aem.56.12.3855-3860.1990 PMC185079

[12] Crossman L, Dow JM. Biofilm formation and dispersal in *Xanthomonas campestris*. Microbes Infect. 2004;6 (6): 623–629. 15158198 10.1016/j.micinf.2004.01.013

[13] da Silva CR, Ferro J, Reinach FC, Farah CS, Furlan LR, Quaggio RB, et al Comparison of the genomes of two *Xanthomonas* pathogens with differing host specificities. Nature. 2002;417 (6887): 459–463. 12024217 10.1038/417459a

[14] Triplett LR, Hamilton JP, Buell CR, Tisserat NA, Verdier V, Zink F, et al Genomic analysis of *Xanthomonas oryzae* isolates from rice grown in the united states reveals substantial divergence from known *X*. *oryzae* pathovars. Appl Environ Microbiol. 2011;77 (12): 3930–3937. 10.1128/AEM.00028-11 21515727 PMC3131649

[15] Qian W, Han Z-J, He C. Two-component signal transduction systems of *Xanthomonas* spp.: a lesson from genomics. Mol Plant-Microbe Interact. 2008;21 (2): 151–161. 10.1094/MPMI-21-2-0151 18184059

[16] Garita-Cambronero J, Sena-Vélez M, Palacio-Bielsa A, Cubero J. Draft genome sequence of *Xanthomonas arboricola* pv. *pruni* strain Xap33, causal agent of bacterial spot disease on almond. Genome Announc. 2014;2 (3): e00440–14. 10.1128/genomeA.00440-14 24903863 PMC4047442

[17] Garita-Cambronero J, Palacio-Bielsa A, López MM, Cubero J. Draft genome sequence for virulent and avirulent strains of *Xanthomonas arboricola* isolated from *Prunus* spp. in Spain. Stand Genomic Sci. 2016;11: 12 10.1186/s40793-016-0132-3 26823958 PMC4730658

[18] Young JM, Park DC, Shearman HM, Fargier E. A multilocus sequence analysis of the genus *Xanthomonas*. Syst Appl Microbiol. 2008;31 (5): 366–377. 10.1016/j.syapm.2008.06.004 18783906

[19] Ignatov AN, Kyrova EI, Vinogradova S V, Kamionskaya AM, Schaad NW, Luster DG. Draft genome sequence of *Xanthomonas arboricola* strain 3004, a causal agent of bacterial disease on barley. Genome Announc. 2015;3 (1): e01572–14. 10.1128/genomeA.01572-14 25700410 PMC4335334

[20] Ibarra Caballero J, Zerillo MM, Snelling J, Boucher C, Tisserat N. Genome sequence of *Xanthomonas arboricola* pv. *corylina*, isolated from Turkish filbert in Colorado. Genome Announc. 2013;1 (3): e00246–13. 10.1128/genomeA.00246-13 23704178 PMC3662818

[21] Higuera G, González-Escalona N, Véliz C, Vera F, Romero J. Draft genome sequences of four *Xanthomonas arboricola* pv. *juglandis* strains associated with walnut blight in Chile. Genome Announc. 2015;3 (5): e01160–15. 10.1128/genomeA.01160-15 26450732 PMC4599091

[22] Pereira UP, Gouran H, Nascimento R, Adaskaveg JE, Goulart LR, Dandekar AM. Complete genome sequence of *Xanthomonas arboricola* pv. *juglandis* 417, a copper-resistant strain isolated from *Juglans regia* L. Genome Announc. 2015;3(5): e01126–15. 10.1128/genomeA.01126-15 26430043 PMC4591315

[23] Harrison J, Grant MR, Studholme DJ. Draft genome sequences of two strains of *Xanthomonas arboricola* pv. *celebensis* isolated from banana plants. Genome Announc. 2016;4(1): e01705–15. 10.1128/genomeA.01705-15 26868395 PMC4751319

[24] Sena-Vélez M. Mecanismos implicados en las etapas iniciales de infección en la cancrosis de los cítricos provocada por Xanthomonas citri subsp. citri. Doctoral dissertation. Politechnic University of Madrid. 2015. Available: http://oa.upm.es/38610/

[25] Mhedbi-Hajri N, Darrasse A, Pigné S, Durand K, Fouteau S, Barbe V, et al Sensing and adhesion are adaptive functions in the plant pathogenic xanthomonads. BMC Evol Biol. 2011;11: 67 10.1186/1471-2148-11-67 21396107 PMC3063832

[26] Darling ACE, Mau B, Blattner FR, Perna NT. Mauve: multiple alignment of conserved genomic sequence with rearrangements. Genome Res. 2004;14 (7): 1394–1403. 15231754 10.1101/gr.2289704 PMC442156

[27] Burch AY, Shimada BK, Browne PJ, Lindow SE. Novel high-throughput detection method to assess bacterial surfactant production. Appl Environ Microbiol. 2010;76 (16): 5363–5372. 10.1128/AEM.00592-10 20562275 PMC2918974

[28] Mattick JS. Type IV pili and twitching motility. Annu Rev Microbiol. 2002;56: 289–314. 12142488 10.1146/annurev.micro.56.012302.160938

[29] Chevance FFV, Hughes KT. Coordinating assembly of a bacterial macromolecular machine. Nat Rev Microbiol. 2008;6 (6): 455–465. 10.1038/nrmicro1887 18483484 PMC5963726

[30] Dunger G, Llontop E, Guzzo CR, Farah CS. The *Xanthomonas* type IV pilus. Curr Opin Microbiol. 2016;30: 88–97. 10.1016/j.mib.2016.01.007 26874963

[31] Filloux A. The underlying mechanisms of type II protein secretion. Biochim Biophys Acta (BBA)—Mol Cell Res. 2004;1694 (1–3): 163–179. 10.1016/j.bbamcr.2004.05.003 15546665

[32] Szczesny R, Jordan M, Schramm C, Schulz S, Cogez V, Bonas U, et al Functional characterization of the Xcs and Xps type II secretion systems from the plant pathogenic bacterium *Xanthomonas campestris* pv *vesicatoria*. New Phytol. 2010;187 (4): 983–1002. 10.1111/j.1469-8137.2010.03312.x 20524995

[33] Vandroemme J, Cottyn B, Baeyen S, De Vos P, Maes M. Draft genome sequence of *Xanthomonas fragariae* reveals reductive evolution and distinct virulence-related gene content. BMC Genomics. 2013;14: 829 10.1186/1471-2164-14-829 24274055 PMC4046712

[34] Subramoni S, Suárez-Moreno ZR, Venturi V. Lipases as pathogenicity factors of plant pathogens In: Timmis KN, editor. Handbook of hydrocarbon and lipid microbiology. Springer; 2010 pp. 3269–3277.

[35] Vorhölter FJ, Schneiker S, Goesmann A, Krause L, Bekel T, Kaiser O, et al The genome of *Xanthomonas campestris* pv. *campestris* B100 and its use for the reconstruction of metabolic pathways involved in xanthan biosynthesis. J Biotechnol. 2008;134 (1–2): 33–45. 10.1016/j.jbiotec.2007.12.013 18304669

[36] Bonas U, Stall RE, Staskawicz B. Genetic and structural characterization of the avirulence gene avrBs3 from *Xanthomonas campestris* pv. *vesicatoria*. Mol Gen Genet. 1989;218 (1): 127–136. 2550761 10.1007/BF00330575

[37] White FF, Potnis N, Jones JB, Koebnik R. The type III effectors of *Xanthomonas*. Mol Plant Pathol. 2009;10 (6): 749–766. 10.1111/j.1364-3703.2009.00590.x 19849782 PMC6640274

[38] Bogdanove AJ, Koebnik R, Lu H, Furutani A, Angiuoli SV., Patil PB, et al Two new complete genome sequences offer insight into host and tissue specificity of plant pathogenic *Xanthomonas* spp. J Bacteriol. 2011;193 (19): 5450–5464. 10.1128/JB.05262-11 21784931 PMC3187462

[39] Guo Y, Figueiredo F, Jones J, Wang N. HrpG and HrpX play global roles in coordinating different virulence traits of *Xanthomonas axonopodis* pv. *citri*. Mol Plant-Microbe Interact. 2011;24 (6): 649–661. 10.1094/MPMI-09-10-0209 21261465

[40] Potnis N, Krasileva K, Chow V, Almeida NF, Patil PB, Ryan RP, et al Comparative genomics reveals diversity among xanthomonads infecting tomato and pepper. BMC Genomics. 2011;12: 146 10.1186/1471-2164-12-146 21396108 PMC3071791

[41] Hajri A, Pothier JF, Le-Saux MF, Bonneau S, Poussier S, Boureau T, et al Type three effector gene distribution and sequence analysis provide new insights into the pathogenicity of plant-pathogenic *Xanthomonas arboricola*. Appl Environ Microbiol. 2012;78 (2): 371–384. 10.1128/AEM.06119-11 22101042 PMC3255760

[42] Li RF, Lu GT, Li L, Su HZ, Feng G, Chen Y, et al Identification of a putative cognate sensor kinase for the two-component response regulator HrpG, a key regulator controlling the expression of the hrp genes in *Xanthomonas campestris* pv. *campestris*. Environ Microbiol. 2014;16 (7): 2053–2071. 10.1111/1462-2920.12207 23906314

[43] Jacobs JM, Pesce C, Lefeuvre P, Koebnik R. Comparative genomics of a cannabis pathogen reveals insight into the evolution of pathogenicity in *Xanthomonas*. Front Plant Sci. 2015;6: 431 10.3389/fpls.2015.00431 26136759 PMC4468381

[44] Pothier JF, Vorhölter FJ, Blom J, Goesmann A, Pühler A, Smits THM, et al The ubiquitous plasmid pXap41 in the invasive phytopathogen *Xanthomonas arboricola* pv. *pruni*: Complete sequence and comparative genomic analysis. FEMS Microbiol Lett. 2011;323 (1): 52–60. 10.1111/j.1574-6968.2011.02352.x 21732961

[45] Burokiene D, Pulawska J. Characterization of *Xanthomonas arboricola* pv. *juglandis* isolated from walnuts in Lithuania. J Plant Pathol. 2012; 94 (1, Supplement): S1.23–S1.27.

[46] Kaluzna M, Pulawska J, Waleron M, Sobiczewski P. The genetic characterization of *Xanthomonas arboricola* pv. *juglandis*, the causal agent of walnut blight in Poland. Plant Pathol. 2014;63 (6): 1404–1416.

[47] Graham JH, Gottwald TR. Research perspectives on eradication of citrus bacterial diseases in Florida. Plant Dis. 1991;75: 1193.

[48] Jalan N, Aritua V, Kumar D, Yu F, Jones JB, Graham JH, et al Comparative genomic analysis of *Xanthomonas axonopodis* pv. *citrumelo* F1, which causes citrus bacterial spot disease, and related strains provides insights into virulence and host specificity. J Bacteriol. 2011;193 (22): 6342–6357. 10.1128/JB.05777-11 21908674 PMC3209208

[49] Stoyanova M, Vancheva T, Moncheva P, Bogatzevska N. Differentiation of *Xanthomonas* spp. causing bacterial spot in Bulgaria based on biolog system. Int J Microbiol. 2014; 2014; 2014: 495476 25197281 10.1155/2014/495476 PMC4150486

[50] Massomo SMS, Nielsen H, Mabagala RB, Mansfeld-Giese K, Hockenhull J, Mortensen CN. Identification and characterisation of *Xanthomonas campestris* pv. *campestris* strains from Tanzania by pathogenicity tests, biolog, rep-PCR and fatty acid methyl ester analysis. Eur J Plant Pathol. 2003;109 (8): 775–789.

[51] Pieretti I, Royer M, Barbe V, Carrere S, Koebnik R, Couloux A, et al Genomic insights into strategies used by *Xanthomonas albilineans* with its reduced artillery to spread within sugarcane xylem vessels. BMC Genomics. 2012;13: 658 10.1186/1471-2164-13-658 23171051 PMC3542200

[52] Noinaj N, Guillier M, Barnard TJ, Buchanan SK. TonB-dependent transporters: regulation, structure, and function. Annu Rev Microbiol. 2010;64: 43–60. 10.1146/annurev.micro.112408.134247 20420522 PMC3108441

[53] Blanvillain S, Meyer D, Boulanger A, Lautier M, Guynet C, Denancé N, et al Plant carbohydrate scavenging through TonB-dependent receptors: A feature shared by phytopathogenic and aquatic bacteria. PLoS One. 2007;2 (2): e224 17311090 10.1371/journal.pone.0000224 PMC1790865

[54] Wadhams GH, Armitage JP. Making sense of it all: bacterial chemotaxis. Nat Rev Mol Cell Biol. 2004;5 (12): 1024–1037. 15573139 10.1038/nrm1524

[55] Moreira LM, Facincani AP, Ferreira CB, Ferreira RM, Ferro MIT, Gozzo FC, et al Chemotactic signal transduction and phosphate metabolism as adaptive strategies during citrus canker induction by *Xanthomonas citri*. Funct Integr Genomics. 2015;15 (2): 197–210. 10.1007/s10142-014-0414-z 25403594

[56] Kearns DB. A field guide to bacterial swarming motility. Nat Rev Microbiol. 2010;8 (9): 634–44. 10.1038/nrmicro2405 20694026 PMC3135019

[57] Malamud F, Torres PS, Roeschlin R, Rigano LA, Enrique R, Bonomi HR, et al The *Xanthomonas axonopodis* pv. *citri* flagellum is required for mature biofilm and canker development. Microbiology. 2011;157 (Pt 3): 819–829. 10.1099/mic.0.044255-0 21109564

[58] Sena-Vélez M, Redondo C, Gell I, Ferragud E, Johnson E, Graham JH, et al Biofilm formation and motility of *Xanthomonas* strains with different citrus host range. Plant Pathol. 2015;64 (4): 767–775.

[59] Shen Y, Chern M, Silva FG, Ronald P. Isolation of a *Xanthomonas oryzae* pv. *oryzae* flagellar operon region and molecular characterization of *flhF*. Mol Plant-Microbe Interact. 2001;14 (2): 204–213 11204784 10.1094/MPMI.2001.14.2.204

[60] Gottig N, Garavaglia BS, Garofalo CG, Orellano EG, Ottado J. A filamentous hemagglutinin-like protein of *Xanthomonas axonopodis* pv. *citri*, the phytopathogen responsible for citrus canker, is involved in bacterial virulence. PLoS One. 2009;4 (2): e4358 10.1371/journal.pone.0004358 19194503 PMC2632755

[61] Köhler T, Curty LK, Barja F, van Delden C, Pechere JC. Swarming of *Pseudomonas aeruginosa* is dependent on cell-to-cell signaling and requires flagella and pili. J Bacteriol. 2000;182 (21): 5990–5996. 11029417 10.1128/jb.182.21.5990-5996.2000 PMC94731

[62] Verstraeten N, Braeken K, Debkumari B, Fauvart M, Fransaer J, Vermant J, et al Living on a surface: swarming and biofilm formation. Trends Microbiol. 2008;16 (10): 496–506. 10.1016/j.tim.2008.07.004 18775660

[63] Sun W, Dunning FM, Pfund C, Weingarten R, Bent AF. Within-species flagellin polymorphism in *Xanthomonas campestris* pv *campestris* and its impact on elicitation of *Arabidopsis* FLAGELLIN SENSING2-dependent defenses. Plant Cell. 2006;18 (3): 764–779. 16461584 10.1105/tpc.105.037648 PMC1383648

[64] Ikeda T, Oosawa K, Hotani H. Self-assembly of the filament capping protein, FliD, of bacterial flagella into an annular structure. J Mol Biol. 1996;259 (4): 679–686. 8683574 10.1006/jmbi.1996.0349

[65] Kim JS, Chang JH, Chung S Il, Yum JS. Molecular cloning and characterization of the *Helicobacter pylori* fliD gene, an essential factor in flagellar structure and motility. J Bacteriol. 1999;181 (22): 6969–6976. 10559162 10.1128/jb.181.22.6969-6976.1999 PMC94171

[66] Craig L, Li J. Type IV pili: paradoxes in form and function. Curr Opin Struct Biol. 2008;18 (2): 267–277. 10.1016/j.sbi.2007.12.009 18249533 PMC2442734

[67] Ray SK, Rajeshwari R, Sharma Y, Sonti R V. A high-molecular-weight outer membrane protein of *Xanthomonas oryzae* pv.*oryzae* exhibits similarity to non-fimbrial adhesins of animal pathogenic bacteria and is required for optimum virulence. Mol Microbiol. 2002;46 (3): 637–647. 12410822 10.1046/j.1365-2958.2002.03188.x

[68] Das A, Rangaraj N, Sonti R V. Multiple adhesin-like functions of *Xanthomonas oryzae* pv. *oryzae* are involved in promoting leaf attachment, entry, and virulence on rice. Mol Plant-Microbe Interact. 2009;22 (1): 73–85. 10.1094/MPMI-22-1-0073 19061404

[69] Ryan RP, Vorhölter FJ, Potnis N, Jones JB, Van Sluys MA, Bogdanove AJ, et al Pathogenomics of *Xanthomonas*: understanding bacterium-plant interactions. Nat Rev Microbiol. 2011;9 (5): 344–355. 10.1038/nrmicro2558 21478901

[70] Cianciotto NP. Type II secretion: A protein secretion system for all seasons. Trends Microbiol. 2005;13 (12): 581–588. 16216510 10.1016/j.tim.2005.09.005

[71] Yun MH, Torres PS, El Oirdi M, Rigano LA, Gonzalez-Lamothe R, Marano MR, et al Xanthan induces plant susceptibility by suppressing callose deposition. Plant Physiol. 2006;141 (1): 178–187. 16531487 10.1104/pp.105.074542 PMC1459321

[72] Katzen F, Ferreiro DU, Oddo CG, Ielmini MV, Becker A, Pühler A, et al *Xanthomonas campestris* pv. *campestris* gum mutants: Effects on xanthan biosynthesis and plant virulence. J Bacteriol. 1998;180 (7): 1607–1617 9537354 10.1128/jb.180.7.1607-1617.1998 PMC107069

[73] Jalan N, Kumar D, Yu F, Jones JB, Graham JH, Wang N. Complete genome sequence of *Xanthomonas citri* subsp. *citri* strain A^w^12879, a restricted-rost-range citrus canker-causing bacterium. Genome Announc. 2013;1 (3): e00235–13. 10.1128/genomeA.00235-13 23682143 PMC3656205

[74] Hall T. BioEdit: An important software for molecular biology. GERF Bull Biosci. 2011;2 (1): 60–61.

[75] Goujon M, McWilliam H, Li W, Valentin F, Squizzato S, Paern J, et al A new bioinformatics analysis tools framework at EMBL-EBI. Nucleic Acids Res. 2010;38 (Web Server issue): W695–W699. 10.1093/nar/gkq313 20439314 PMC2896090

[76] Posada D. jModelTest: Phylogenetic model averaging. Mol Biol Evol. 2008;25 (7): 1253–6. 10.1093/molbev/msn083 18397919

[77] Tamura K, Peterson D, Peterson N, Stecher G, Nei M, Kumar S. MEGA5: Molecular evolutionary genetics analysis using maximum likelihood, evolutionary distance, and maximum parsimony methods. Mol Biol Evol. 2011;28 (10): 2731–2739. 10.1093/molbev/msr121 21546353 PMC3203626

[78] Posada D, Buckley TR. Model selection and model averaging in phylogenetics: advantages of akaike information criterion and bayesian approaches over likelihood ratio tests. Syst Biol. 2004;53 (5): 793–808. 15545256 10.1080/10635150490522304

[79] Seemann T. Prokka: rapid prokaryotic genome annotation. Bioinformatics. 2014;30 (14): 2068–2069. 10.1093/bioinformatics/btu153 24642063

[80] Page AJ, Cummins CA, Hunt M, Wong VK, Reuter S, Holden MTG, et al Roary: rapid large-scale prokaryote pan genome analysis. Bioinformatics. 2015;31 (22): 3691–3693. 10.1093/bioinformatics/btv421 26198102 PMC4817141

[81] Krogh A, Larsson B, von Heijne G, Sonnhammer EL. Predicting transmembrane protein topology with a hidden Markov model: application to complete genomes. J Mol Biol. 2001;305 (3): 567–580. 11152613 10.1006/jmbi.2000.4315

[82] Petersen TN, Brunak S, von Heijne G, Nielsen H. SignalP 4.0: discriminating signal peptides from transmembrane regions. Nat Methods. 2011;8 (10): 785–786. 10.1038/nmeth.1701 21959131

[83] Tatusov RL. The COG database: a tool for genome-scale analysis of protein functions and evolution. Nucleic Acids Res. 2000;28 (1): 33–36. 10592175 10.1093/nar/28.1.33 PMC102395

[84] Marchler-Bauer A, Derbyshire MK, Gonzales NR, Lu S, Chitsaz F, Geer LY, et al CDD: NCBI’s conserved domain database. Nucleic Acids Res. 2014;43 (Database issue): D222–6. 10.1093/nar/gku1221 25414356 PMC4383992

[85] Katoh K, Misawa K, Kuma K, Miyata T. MAFFT: a novel method for rapid multiple sequence alignment based on fast Fourier transform. Nucleic Acids Res. 2002;30 (14): 3059–3066. 12136088 10.1093/nar/gkf436 PMC135756

[86] Stamatakis A. RAxML version 8: a tool for phylogenetic analysis and post-analysis of large phylogenies. Bioinformatics. 2014;30 (9): 1312–1313. 10.1093/bioinformatics/btu033 24451623 PMC3998144

[87] Huson DH, Richter DC, Rausch C, Dezulian T, Franz M, Rupp R. Dendroscope: An interactive viewer for large phylogenetic trees. BMC Bioinformatics. 2007;8: 460 18034891 10.1186/1471-2105-8-460 PMC2216043

[88] Vernière C, Pruvost O, Civerolo EL, Gambin O, Jacquemoud-Collet JP, Luisetti J. Evaluation of the biolog substrate utilization system to identify and assess metabolic variation among strains of *Xanthomonas campestris* pv. *citri*. Appl Environ Microbiol. 1993;59 (1): 243–249 16348849 10.1128/aem.59.1.243-249.1993 PMC202085

[89] Maharjan RP, Seeto S, Ferenci T. Divergence and redundancy of transport and metabolic rate-yield strategies in a single *Escherichia coli* population. J Bacteriol. 2007;189 (6): 2350–2358. 17158684 10.1128/JB.01414-06 PMC1899394

[90] National Center for Biotechnology Information (NCBI). How to: Find a homolog for a gene in another organism. 2016. Available: http://www.ncbi.nlm.nih.gov/guide/howto/find-homolog-gene/

[91] Pothier JF, Smits THM, Blom J, Vorhölter FJ, Goesmann A, Pühler A, et al Complete genome sequence of the stone fruit pathogen *Xanthomonas arboricola* pv. *pruni*. Phytopathology. 2011;101: S144–S145.

[92] Blocker A, Komoriya K, Aizawa S-I. Type III secretion systems and bacterial flagella: insights into their function from structural similarities. Proc Natl Acad Sci USA. 2003;100 (6): 3027–3030. 12631703 10.1073/pnas.0535335100 PMC152238

[93] Büttner D, Bonas U. Regulation and secretion of *Xanthomonas* virulence factors. FEMS Microbiol Rev. 2010;34 (2): 107–133. 10.1111/j.1574-6976.2009.00192.x 19925633

[94] Abby SS, Rocha EPC. The non-flagellar type III secretion system evolved from the bacterial flagellum and diversified into host-cell adapted systems. PLoS Genet. 2012;8 (9): e1002983 10.1371/journal.pgen.1002983 23028376 PMC3459982

[95] He Y-W, Zhang L-H. Quorum sensing and virulence regulation in *Xanthomonas campestris*. FEMS Microbiol Rev. 2008;32 (5): 842–857. 10.1111/j.1574-6976.2008.00120.x 18557946

[96] Nascimento R, Gouran H, Chakraborty S, Gillespie HW, Almeida-Souza HO, Tu A, et al The type II secreted lipase/esterase LesA is a key virulence factor required for *Xylella fastidiosa* pathogenesis in grapevines. Sci Rep. 2016;6: 18598 10.1038/srep18598 26753904 PMC4709584

[97] Alikhan NF, Petty NK, Ben Zakour NL, Beatson SA. BLAST Ring Image Generator (BRIG): simple prokaryote genome comparisons. BMC Genomics. 2011;12: 402 10.1186/1471-2164-12-402 21824423 PMC3163573

[98] Grant JR, Stothard P. The CGView server: a comparative genomics tool for circular genomes. Nucleic Acids Res. 2008; 36(Web Server issue): W181–W184: 181–184. 10.1093/nar/gkn179 18411202 PMC2447734

