# Supplementary figures and images for: Comparative Genomic and Phenotypic Characterization of Pathogenic and Non-Pathogenic Strains of Xanthomonas arboricola Reveals Insights into the Infection Process of Bacterial Spot Disease of Stone Fruits
Source: PLoS One. 2016 Aug 29;11(8):e0161977. doi: 10.1371/journal.pone.0161977 (PMC5003339; doi:10.1371/journal.pone.0161977)

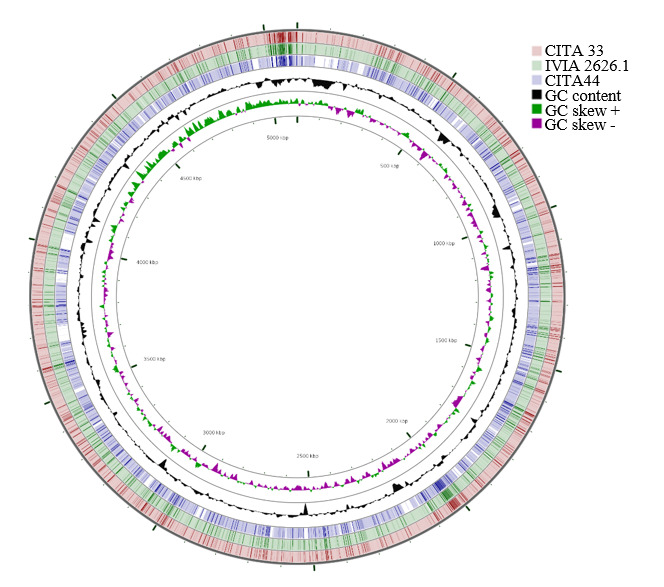

Supplement: S1 Fig — Genome sequences were compared against each other based on Blastn results and represented as a circular map using the CGview tool. From outside to center: strain CITA 33, strain IVIA 2626.1, strain CITA 44, GC content, GC skew + and GC skew -. (TIF) [file pone.0161977.s001.tif]

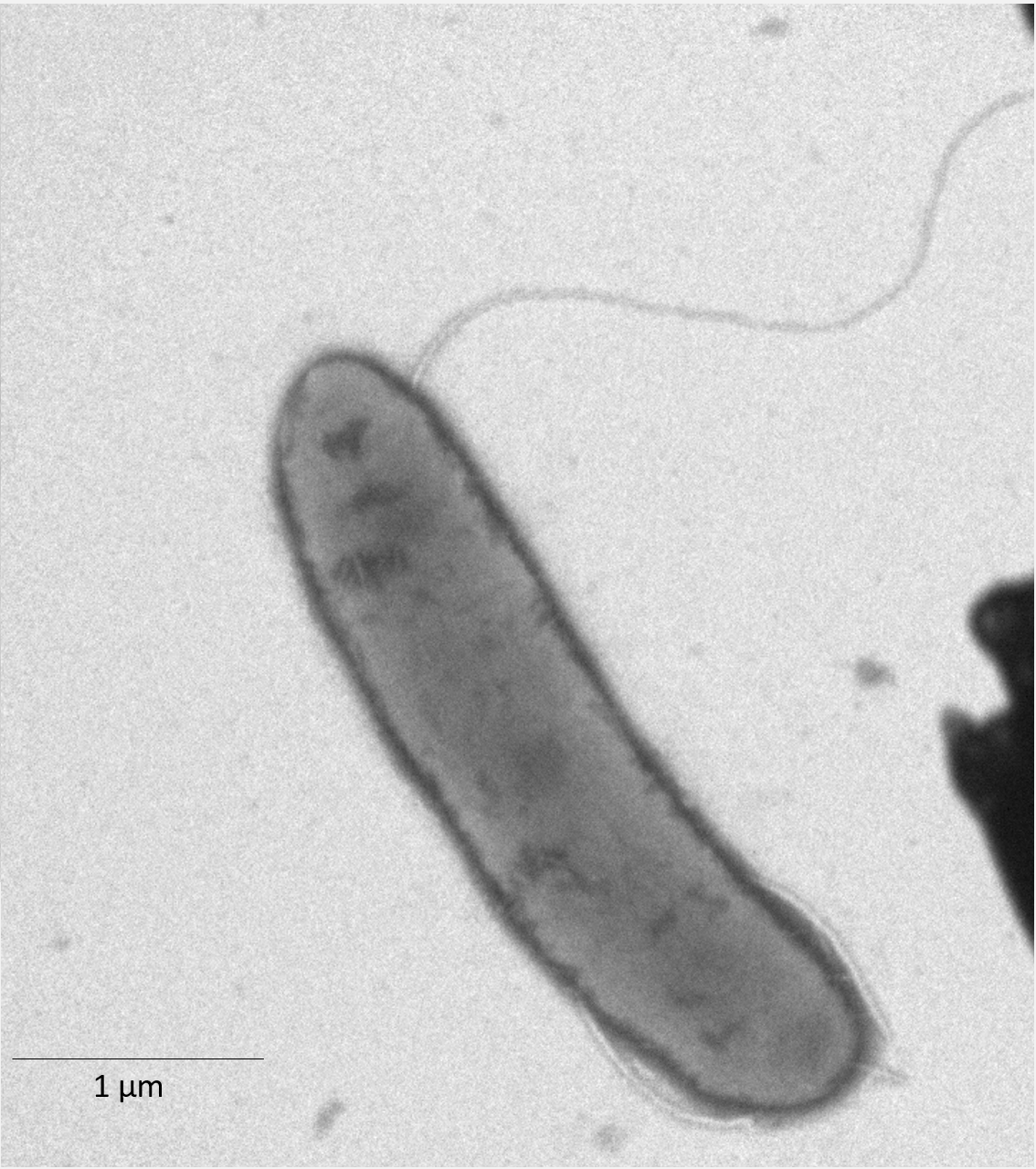

Supplement: S2 Fig — Negative staining showed monoflagellated cell from the edge of the dendritic swarming colony 24 hpi in 0.5% PYM agar plates. (TIF) [file pone.0161977.s002.tif]

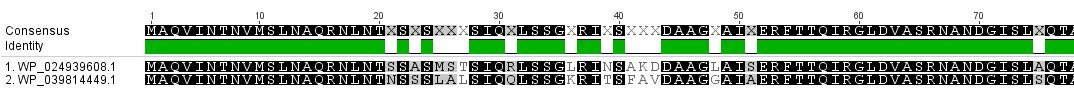

Supplement: S3 Fig — Sequence alignment, performed using ClustalW, showed the amino acid change in the position number 43 of the amino terminal region of FliC. (TIF) [file pone.0161977.s003.tif]

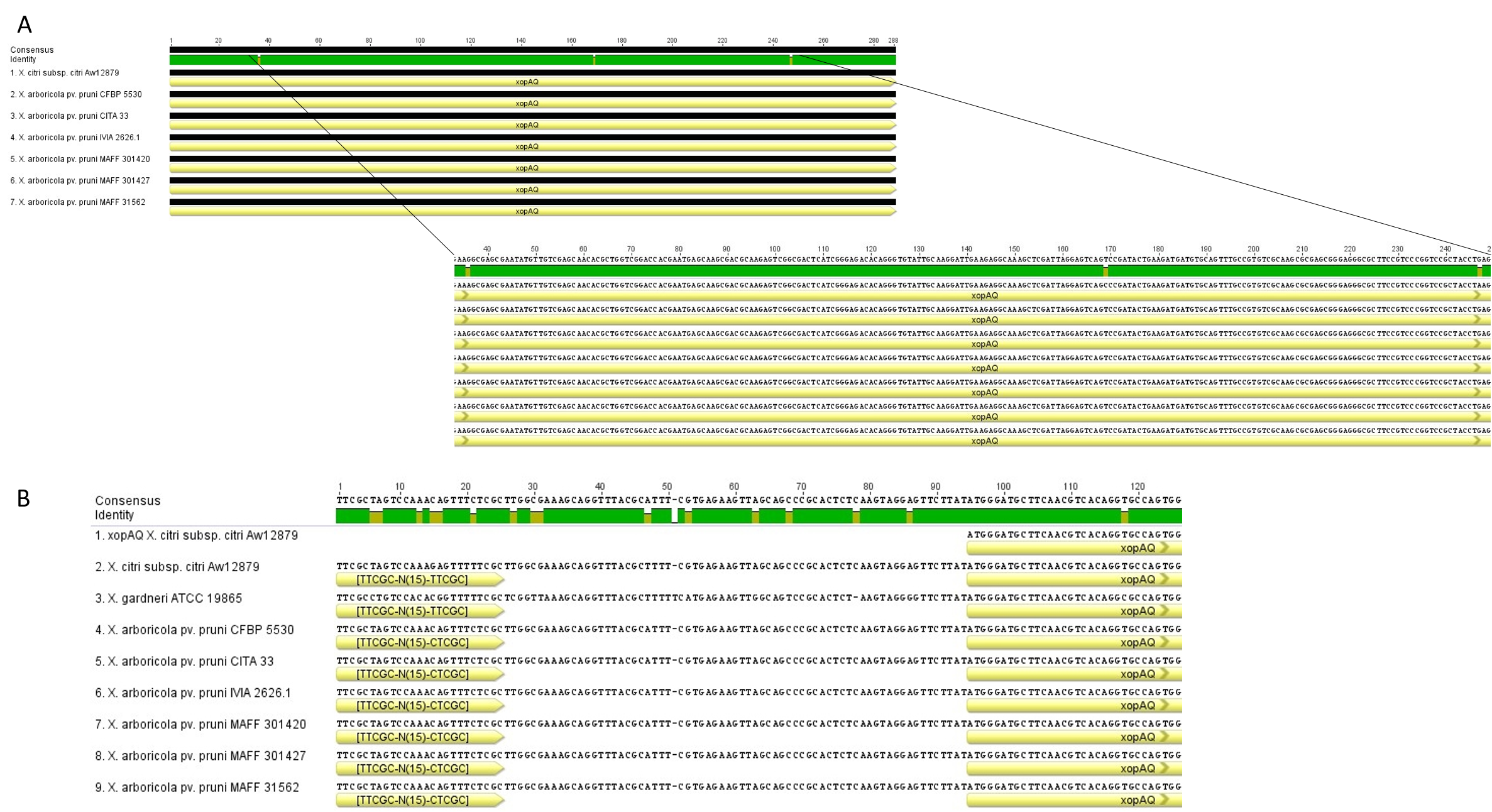

Supplement: S4 Fig — Sequence alignment, performed using ClustalW, showed a slight variation among X. arboricola and other Xanthomonas (A) as well as a variant in PIP-box sequence (B). (TIF) [file pone.0161977.s004.tif]
